# The Maternal-Effect Gene *cellular island* Encodes Aurora B Kinase and Is Essential for Furrow Formation in the Early Zebrafish Embryo

**DOI:** 10.1371/journal.pgen.1000518

**Published:** 2009-06-19

**Authors:** Taijiro Yabe, Xiaoyan Ge, Robin Lindeman, Sreelaja Nair, Greg Runke, Mary C. Mullins, Francisco Pelegri

**Affiliations:** 1 Laboratory of Genetics, University of Wisconsin – Madison, Madison, Wisconsin, United States of America; 2 Department of Cell and Developmental Biology, University of Pennsylvania Medical School, Philadelphia, Pennsylvania, United States of America; Stanford University School of Medicine, United States of America

## Abstract

Females homozygous for a mutation in *cellular island (cei)* produce embryos with defects in cytokinesis during early development. Analysis of the cytoskeletal events associated with furrow formation reveal that these defects include a general delay in furrow initiation as well as a complete failure to form furrow-associated structures in distal regions of the blastodisc. A linkage mapping-based candidate gene approach, including transgenic rescue, shows that *cei* encodes the zebrafish Aurora B kinase homologue. Genetic complementation analysis between the *cei* mutation and *aurB* zygotic lethal mutations corroborate gene assignment and reveal a complex nature of the maternal-effect *cei* allele, which appears to preferentially affect a function important for cytokinesis in the early blastomeres. Surprisingly, in *cei* mutant embryos a short yet otherwise normal furrow forms in the center of the blastodisc. Furrow formation is absent throughout the width of the blastodisc in *cei* mutant embryos additionally mutant for *futile cycle*, which lack a spindle apparatus, showing that the residual furrow signal present in *cei* mutants is derived from the mitotic spindle. Our analysis suggests that partially redundant signals derived from the spindle and astral apparatus mediate furrow formation in medial and distal regions of the early embryonic blastomeres, respectively, possibly as a spatial specialization to achieve furrow formation in these large cells. In addition, our data also suggest a role for Cei/AurB function in the reorganization of the furrow-associated microtubules in both early cleavage- and somite-stage embryos. In accordance with the requirement for *cei/aurB* in furrow induction in the early cleavage embryo, germ plasm recruitment to the forming furrow is also affected in embryos lacking normal *cei/aurB* function.

## Author Summary

The molecular details driving the splitting of cells in two during cell division, known as cytokinesis, remain incompletely understood, as is the coordination of this process with events that pattern the early animal embryo. The dearth of our knowledge in this subject is particularly evident in the case of vertebrate embryonic development. This is in large part because of challenges associated with the identification and characterization in vertebrate systems of the responsible genes, which are expressed during oogenesis. We have carried out genetic screens to identify in an unbiased manner maternal-effect genes required for early zebrafish development. Here we report the molecular genetic analysis of one such gene, *cellular island*. We show that *cellular island* encodes the chromosomal passenger complex protein Aurora B kinase and that its function is essential for furrow induction and maturation in the zebrafish embryo. Genetic analysis involving mutations in this gene and a maternal-effect gene required for spindle formation shows the presence of spatial and temporal specializations in furrow induction signals and *cellular island/aurora B kinase* function in the early embryo.

## Introduction

Following fertilization, an increase in cell number through cell division characterizes the initiation of early embryonic development. Cell division itself, and specifically cytokinesis, the physical process that divides a cell into two daughter cells, remains incompletely understood. Early stages of cytokinesis involve the specification of the cleavage site midway between the spindle poles [1],[2]. Recently, it has been proposed that signals from both astral and spindle microtubules act redundantly in furrow initiation [3]–[5]. In these studies, astral microtubules have been proposed to induce furrow initiation while a subsequent spindle midzone-derived signal further promotes furrow formation.

The Aurora B kinase (AurB) is thought to be a crucial factor in multiple processes in cell division, including furrow formation during cytokinesis. AurB, together with other factors such as Incenp, Survivin and CSC-1, is a component of the chromosomal passenger complex, which is localized to centrosomes prior to mitosis but becomes localized to centromeres in metaphase and to the spindle midzone after chromatid segregation in anaphase (reviewed in [6]). Independent of its accumulation at the furrow via the spindle midzone, AurB is also delivered to the prospective cleavage site along astral microtubules [7],[8]. Chromosomal passenger protein function has been implicated in the localization to the central spindle of centralspindlin, a complex comprising the kinesin subfamily member Mklp1 (also known as ZEN-4 and Pavarotti) and the Rho family GTPase-activating protein RacGAP (also known as CYK-4, RacGAP50C and MgcRacGAP; reviewed in [9]). Centralspindlin is thought to have a dual function in the initiation of cytokinesis. On one hand, the bundling activity of Mklp1 helps promote the stability of the midzone microtubule apparatus. On the other hand, RacGAP promote changes in actomyosin dynamics that result in the formation of the contractile ring and furrow constriction.

In early zebrafish embryos, cytokinesis is associated with cytoskeletal rearrangements, one of which involves the formation of the contractile ring apparatus [10]. Furrow initiation is also associated with the assembly of the furrow microtubule array (FMA), a structure that consists of microtubules originally organized parallel to each other and perpendicular to the plane of cleavage [10]–[13]. The FMA is thought to be functionally analogous to the bundles of midzone microtubules observed in other animal and plant systems [14]. During furrow maturation a second, myosin II-dependent phase of cytokinesis occurs which includes the movement of cortical cell adhesion junction components towards the furrow plane and the translocation of FMA tubules along the furrow plane towards the distal ends of the furrow [10]. In addition, during furrow maturation, localized exocytosis mediated by FMA tubules has been suggested to have a role in the formation of the new membrane septum between daughter cells [12].

In the animal embryo, the earliest cellular divisions are often coordinated with the segregation of localized maternal determinants that contribute to cell fate diversification. One of the earliest decisions commonly involves the specification of the germ line, via the acquisition of the germ plasm, a specialized cytoplasm that contains specific mRNA and protein products [15]. In zebrafish, the segregation of germ plasm components is intimately linked to the process of cytokinesis, as ultrastructurally defined germ plasm forms at the furrows of the first and second cleavage divisions [16]. Several mRNAs, such as those for the genes *vasa* [17], *dead end* [18], *nanos* [19], *daz-like* [20], *bruno-like* [20], and *askopos* [21], as well as Brul protein [22], have been shown to be components of the zebrafish germ plasm. A subclass of germ plasm mRNAs, including *vasa*, *dead end* and *nanos*, is present in distinct aggregates at the blastodisc cortex during the first cell cycle [23]. These mRNAs undergo a complex segregation pattern immediately prior to and during furrow formation, including their pre-aggregation during the first cell cycle, their recruitment as rod-like structures at the incipient furrows, and the subsequent enrichment and compaction of the recruited germ plasm to the distal end of the maturing furrow [10],[13],[16],[17],[23]. The cellular mechanism involved in the recruitment of germ plasm aggregates during furrow formation remains poorly understood.

Recent efforts have led to the isolation of recessive maternal-effect mutations in the zebrafish that affect a variety of early developmental processes, including the process of cytokinesis [24]–[28]. One of these mutations, in the gene *cellular island (cei)* was originally identified as essential for proper early cleavage divisions [25]. Here, we show that the *cei* maternal-effect mutation results in an aberrant allele of the zebrafish *aurora B kinase (aurB) gene*. Our data show that *cei/aurB* function is essential for furrow initiation in the blastomeres of the early embryo, and that different signals, mediated by spindle midzone and astral microtubules respectively, are important for the induction in medial vs. distal regions in the large cells of the early embryo. In addition, we show a role for AurB function in the reorganization of the microtubule apparatus at the furrow. Concordant with the requirement for *cei/aurB* in furrow induction, germ plasm recruitment to the cleavage furrows during the first two cell cycles is also affected in *cei/aurB* mutant embryos.

## Results

### A Maternal-Effect Mutation in *cellular island* Results in Embryonic Lethality Caused by Defects in Furrow Formation

A mutation in the gene *cellular island* was originally isolated by its associated maternal-effect phenotype in the blastula stage zebrafish embryo. The initial analysis of this mutation showed that females carrying the *cei* mutation are viable and lack any obvious visible phenotype. However, all embryos from such mutant females, which we refer to as *cei* mutant embryos, are inviable, often exhibiting a mass or “island” of cells sitting atop an abnormally expanded syncytial region [25].

**Figure 1:**
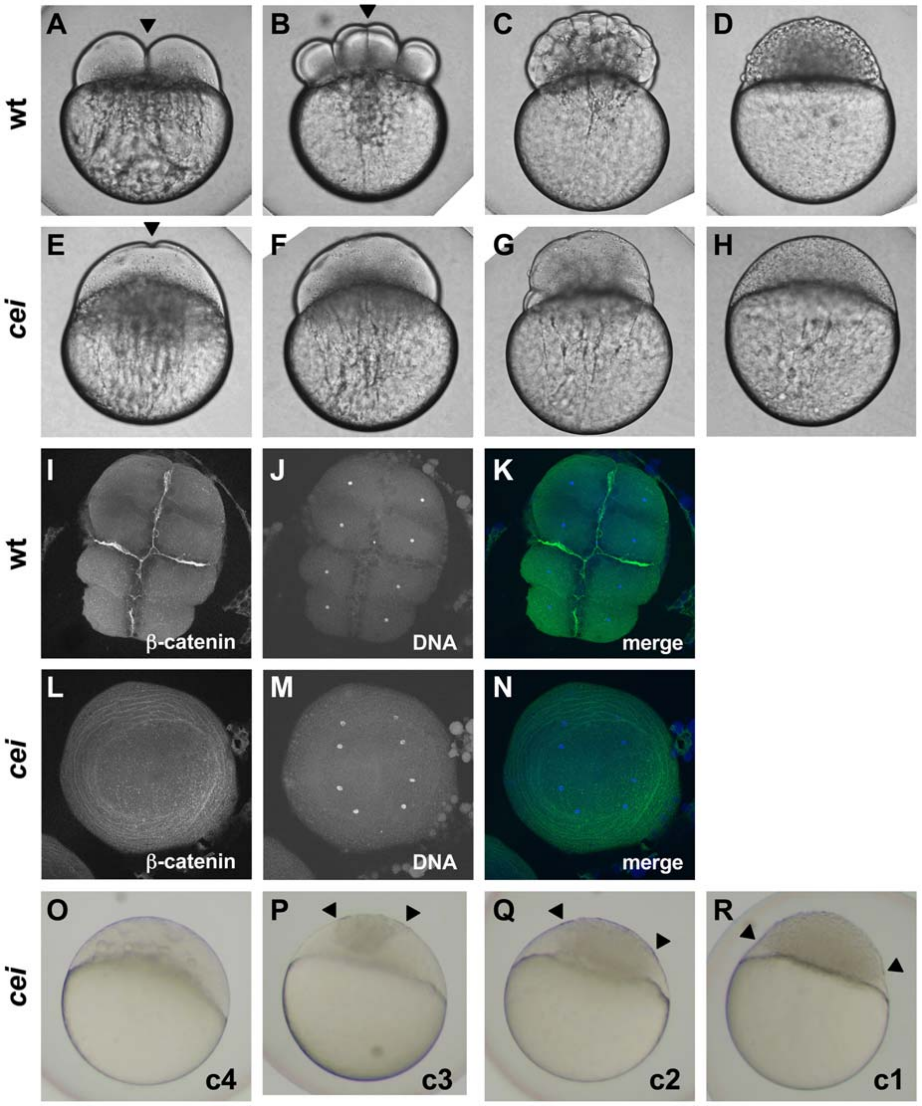
Embryos from *cei* homozygous females exhibit defects in cytokinesis. Cytokinesis defects in live (A–H, O–R) and fixed (I–N) wild-type (A–D, I–K) and *cei* mutant (E–H, L–R) embryos. (A–H) Side views of live wild-type (A–D) and maternally mutant *cei* (E–H) embryos at time points equivalent to (in wild-type embryos) the 2-cell (A,E), 8-cell (B,F), 64-cell (C,G) and 1,000-cell (D,H) stages. Maternally mutant *cei* embryos exhibit a rudimentary furrow (arrowhead in E) and form syncytial embryos (H). (I–N) Animal views of fixed wild-type (I–K) and maternally mutant *cei* (L–N) embryos, labeled with an antibody against ß-catenin, a component of cell adhesion junctions present in mature furrows (I,L) and the DNA dye DAPI (J,M). Merged images shown in (K,N). (O–R) Side views of live embryos at the 1,000 cell stage, showing the phenotypic range of maternally mutant *cei* embryos. Embryos exhibit various degrees of aberrant syncytium formation. Images are representative for the categories presented in Table 1: C4 (most severe – O), C3 (P), C2 (Q), C1 (least severe – R). Arrowheads show the limits of a single cellularized region that typically sits atop the syncytial region.

**Figure 2:**
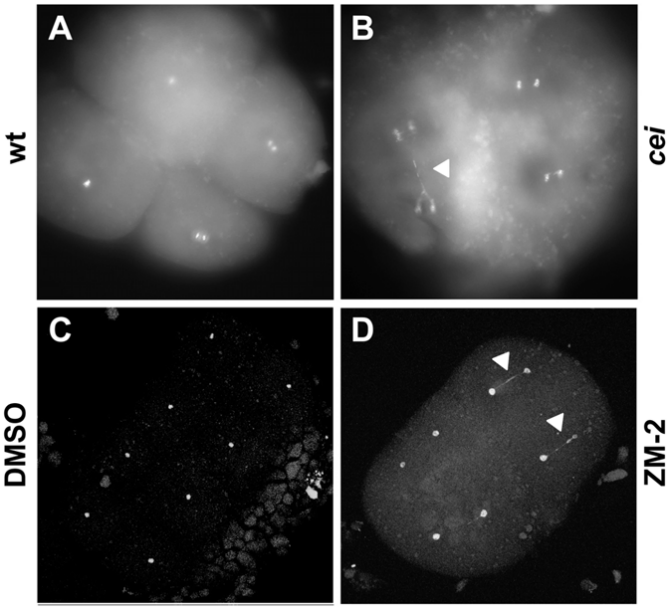
DNA segregation defects in embryos with reduced *cei/aurB* function. Animal views of fixed embryos labeled with DAPI. (A–B) Wild-type (A) and maternally mutant *cei* (B) embryos at 60 min p.f. (C–D) Solvent- (C) and ZM2- (D) treated embryos at 75 min p.f. Arrowheads indicate DNA bridges.

The expanded syncytium typically observed in *cei* mutant embryos is characteristic of early zebrafish embryos with defects in cell division [13],[25],[29],[30], which suggested that *cei* function is required for this process. In wild-type embryos, cells during the early cleavage stages divide by invagination of the membrane at the site of furrow formation (shown for the first cell division in Figure 1A, arrowhead), which matures into an adhesive membrane septum during subsequent divisions (arrowhead in Figure 1B shows the mature septum in the first cleavage furrow, at the 8-cell stage). Continuing cellular cleavage results in the normally cellularized blastula (Figure 1C and 1D). *cei* mutant embryos typically lack the normal ingression of the forming furrow, showing instead a slight indentation where a furrow would normally develop (arrowhead in Figure 1E). Consequently, *cei* mutants develop largely as syncytial embryos (Figure 1F–1H). Labeling of 8-cell stage fixed embryos to detect ß-catenin, a component of cell adhesive junctions at the mature furrow [12], and DNA corroborates the absence of adhesive membrane formation in *cei* mutant embryos, and shows that nuclear division proceeds normally (Figure 1L–1N, compare to wild-type embryos in Figure 1I–1K). Thus, the *cei* mutation appears to interfere with furrow formation during cytokinesis. Although nuclear division appears normal in most *cei* mutant embryos (Figure 1M, indistinguishable from wild-type in Figure 1J), a fraction of mutant embryos exhibit DNA bridges between daughter nuclei indicative of DNA segregation defects (Figure 2B, compare to Figure 2A). Thus, the *cei* mutation interferes with furrow formation during cytokinesis and *cei* function may be additionally required for proper chromosome segregation.

We noted that the severity of the maternal-effect cellularization defect varied in different females within families carrying the *cei* mutation. While some females showed a highly penetrant defect, in which all embryos lacked proper furrow formation in the first and subsequent cell cycles, clutches from other females showed much more variable defects (Table 1; the severity of these defects has been classified according to the extent of cellularization in the 1,000 cell stage embryo, as shown in Figure 1O–1R). Genotyping of females using previously identified linked markers (see Materials and Methods) showed that the severity of the maternal-effect phenotype closely corresponds to the maternal genotype: females homozygous for the *cei* mutation produced strongly affected clutches in which furrows do not form in all or most embryos, while females heterozygous for the mutation produced clutches with cellularization defects varying from weak to undetectable (Table 1). Thus, the *cei* allele results in a highly penetrant recessive maternal-effect phenotype, as well as a partially dominant maternal-effect that leads to cellularization phenotypes of reduced penetrance.

**Table 1:** The maternal-effect *cei* mutation results in a highly penetrant recessive cellularization defect and a partially penetrant dominant phenotype.

Maternal Genotype^1^	Cellularization phenotype (%)^2^ (least severe) (most severe)					n
	normal	c1	c2	c3	c4	
*+/+*	100	0	0	0	0	133
*+/+*	100	0	0	0	0	236
*cei/+*	86	14	0	0	0	65
*cei/+*	56	18	16	8	1	293
*cei/+*	74	18	6	2	0	136
*cei/+*	98	1	0.5	0	0.5	193
*cei/+*	95	5	0	0	0	40
*cei/cei*	0	0	0	0	100	111
*cei/cei*	0	0	0	0	100	160
*aurB^hi1045^/+* 3	100	0	0	0	0	2156
*aurB^hi3986^*/+	100	0	0	0	0	128

1 Genotype of different sibling adult females generating the clutch, mated to wild-type males.

2 Percent of embryos exhibiting cellularization defects of various severities at the 1,000-cell stage, according to the classification presented in Figure 1O–R.

3 Data for *aurB^hi1045^/+* females is pooled from clutches from 11 different females.

**Figure 3:**
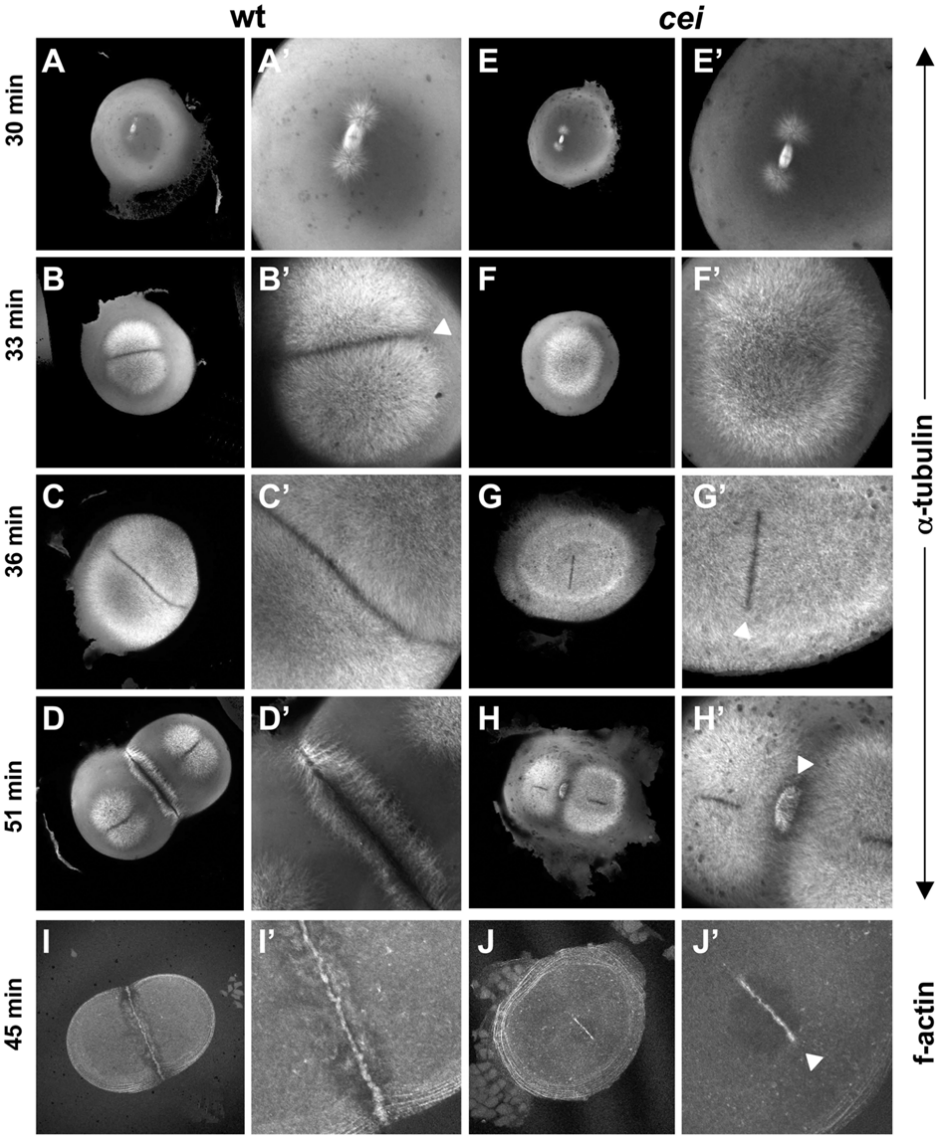
*cei* mutant embryos have defects in the induction of furrow associated structures. (A–H) Animal views of fixed wild-type (A–D) and mutant (E–H) embryos synchronized by in vitro fertilization and fixed at the indicated time points. Initiation of furrow formation (arrowhead in B′) is delayed in mutant embryos (F′), and furrow associated structures form only in medial region in the mutant (arrowheads in G′,H′). (I,J) Fixed wild-type (I) and mutant (J) embryos labeled with fluorescent phalloidin, showing the formation of a truncated furrow in the center of the blastodisc (arrowhead in J′).

**Figure 4:**
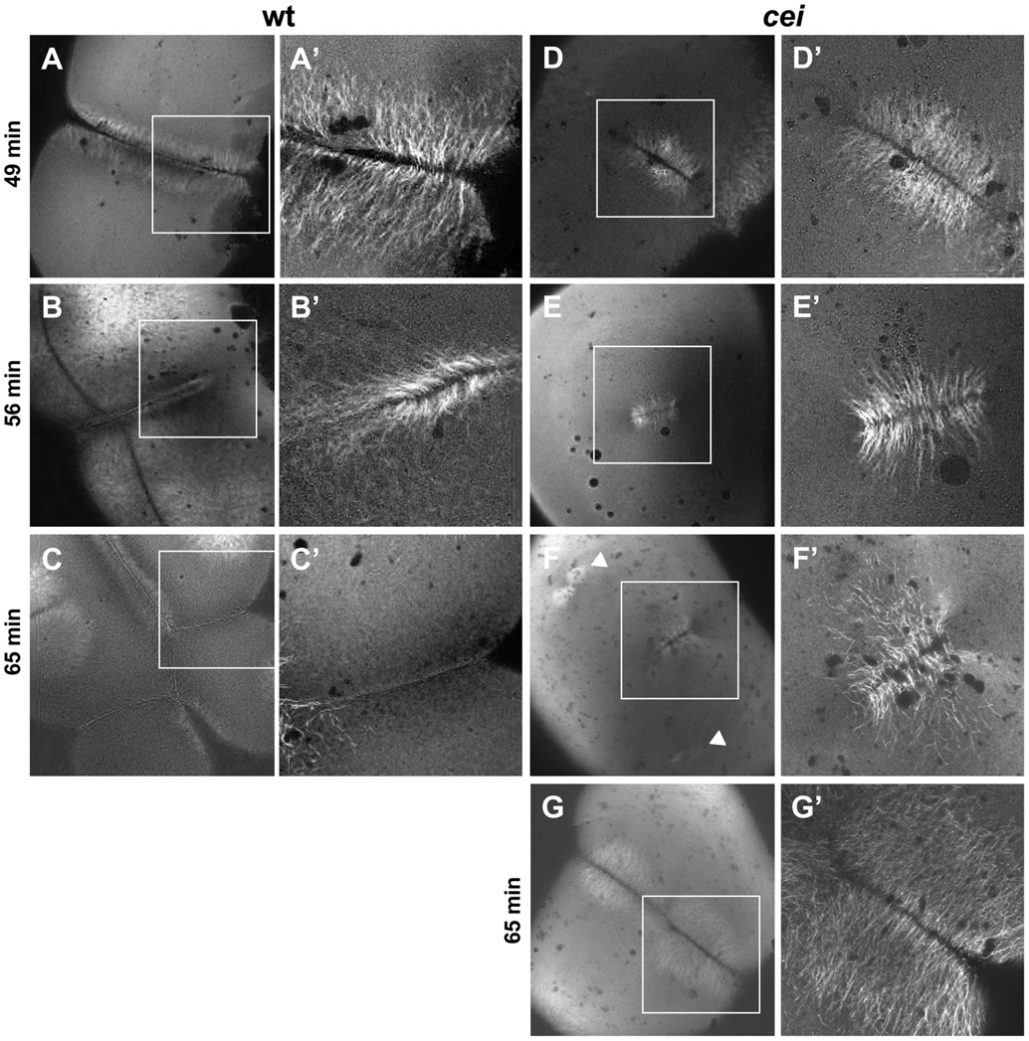
Defective cytoskeletal dynamics in *cei/aurB* mutant embryos. (A–G) High magnification images of wild-type (A–C) and *cei* mutant (D–G) embryos fixed at the indicated time points and labeled with an anti-α-tubulin antibody. Images show the FMA structure, which after formation of the furrow (A) becomes enriched in the distal region of the wild-type embryo (B) and eventually disassembles (C). In maternal *cei* mutants, the truncated FMA forms in the center of the blastodisc (D) and neither translocates distally nor becomes disassembled (E,F). Arrowheads in (F) indicate FMA remnants corresponding to the second cleavage planes. A rare maternal *cei* mutant embryo with a weaker phenotype (G) showing that defects in FMA reorganization and disassembly can be observed even when the furrow encompasses the entirety of the blastodisc.

To better understand the basis of the cellularization defect observed in *cei* mutants, we visualized furrow-associated structures in wild-type and mutant embryos synchronized by in vitro fertilization. We first analyzed the microtubule-based cytoskeleton (Figure 3A–3H). As previously described [12],[13],[23],[31], wild-type embryos exhibit a bipolar spindle and associated astral microtubules immediately prior to furrow formation (30 min postfertilization (p.f.); Figure 3A). During furrow initiation (33 min p.f.) astral microtubules in wild-type embryos cover most of the blastodisc (Figure 3B), leaving a distinct microtubule-free zone in the region corresponding to the initiating furrow (arrowhead in Figure 3B′). At 36 min p.f., astral microtubules begin to form the FMA (Figure 3C). The FMA persists during furrow maturation (51 min p.f.; Figure 3D), which temporally overlaps the initiation of the second cell division cycle [10],[12],[13]. In *cei* mutant embryos, the bipolar spindle and associated asters appear to form normally and grow at a normal rate immediately prior to furrow formation (30 min p.f., Figure 3E and 3F), although we can not rule out subtle changes in their structure or dynamics. However, mutant embryos show striking differences to wild-type beginning at furrow initiation. At 33 min p.f., although microtubule asters in *cei* mutant embryos increase in length as in wild-type embryos, they do not yet form a distinct microtubule-free zone at the forming furrow (Figure 3F). This lack of a microtubule-free region at the furrow is temporary, as a clearing of astral microtubules corresponding to the initiating furrow flanked by apparently normal FMA tubules appears at 36 min p.f. (Figure 3G, arrowhead in Figure 3G′). However, both the microtubule-free region corresponding to the initiating furrow and FMA tubules are completely absent in distal regions of the blastodisc. Furrows of subsequent cell cycles show similar defects (Figure 3H and data not shown, see also Figure 4F).

We also analyzed *cei* mutant embryos by labeling fixed embryos to detect f-actin, which becomes recruited to the forming furrow as part of the contractile ring apparatus [10]. Similar to the case of FMA formation, f-actin accumulation at the furrow only occurs in a shortened region centered in the middle of the blastodisc, and is absent in more distal regions (Figure 3J, compare to Figure 3I). Thus, the rudimentary furrows formed in *cei* mutant embryos correspond to a shortened medially-located furrow and reflect a lack of furrow formation in the distal regions of the blastodisc.

### *cellular island* Mutants Exhibit Defects in Microtubule Reorganization During Furrow Maturation

Further analysis of microtubules revealed that, in addition to the furrow initiation phenotype, *cei* mutants exhibit defects in FMA reorganization during furrow maturation (Figure 4; [10],[12],[13]). In wild-type embryos, tubules of the FMA are arranged parallel to each other and perpendicular to the furrow when they are recruited to the forming furrow (Figure 4A). During furrow maturation, tubules become enriched distally, acquiring a tilted angle with respect to the furrow itself, such that tubules abutting the furrow form V-shaped structures pointing towards the furrow distal end (Figure 4B). At furrow completion, FMA tubules disassemble (Figure 4C). In *cei* mutant embryos, FMA tubules become recruited with their normal conformation (perpendicular to the furrow) in the shortened medial region of the blastomere (Figure 4D). However, these tubules fail to undergo the rearrangements and disassembly observed in wild-type embryos at later stages of furrow development (Figure 4E and 4F), maintaining instead their original arrangement at a time when the FMA is fully disassembled in control wild-type embryos. This defect in FMA reorganization can also be observed in rare *cei* mutant embryos that exhibit a furrow of a normal length (Figure 4G), suggesting that the absence of FMA reorganization in *cei* mutants is not a secondary consequence of the shortened furrow normally observed in these mutants. Our observations are consistent with a role for *cei* in cytoskeletal reorganization during furrow maturation.

### *cellular island* Encodes the Zebrafish *Aurora B Kinase* Homologue

Linkage analysis of polymorphic DNA markers defined an approximately 16 cM region on chromosome 14 that contains the *cellular island* locus (Figure S1A). A set of markers included within this region and located at position 2.30 cM in the MGH meiotic map were fully linked to the *cei* mutation in all 351 tested meioses. Analysis of the available zebrafish genomic databases revealed the presence of a *serine/threonine kinase a* (*stka*) gene with similarity to aurora kinases in the vicinity of these markers. Sequence comparison between zebrafish *stka* and other aurora kinase genes shows that *stka* is a likely homologue of *aurora B kinase* (*aurB*; Figure S1B). Thus, we hereafter refer to zebrafish *stka* as zebrafish *aurB*. Sequencing of the *aurB* cDNA derived from mutant and wild-type embryos revealed a predicted amino acid change from a Valine to a Methionine at position 271 of the protein. The presence of a Valine at this position is absolutely conserved in all analyzed aurora B kinase homologues, including the fission yeast *Schizosaccaromyces pombe*, *Arabidopsis*, *Caenorhabditis elegans*, *Drosophila* and humans, but is not conserved in the more distantly related Polo like kinase 4 (Figure S1C). This high level of amino acid conservation amongst Aurora kinase family genes suggests that a Valine at this position is crucial for their normal function.

**Figure 5:**
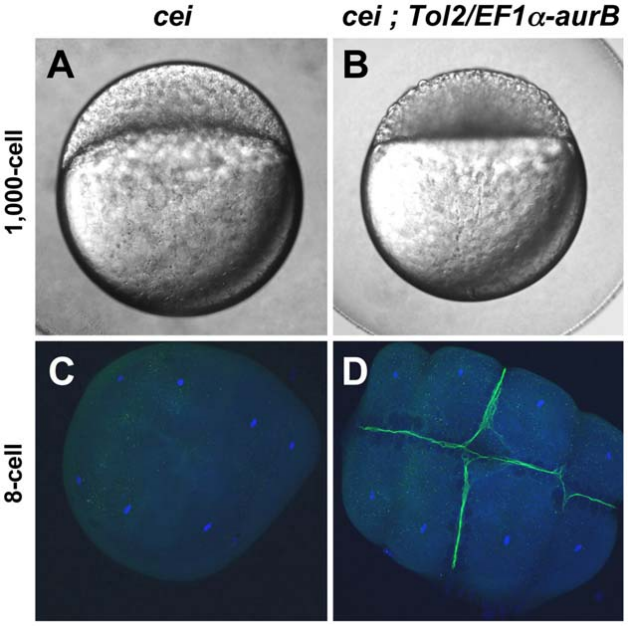
Maternal expression of wild-type aurora B kinase rescues the cytokinesis defects in *cei* mutant embryos. Embryos from *cei/cei* homozygous mutant females show a strong syncytial phenotype at the 1,000 cells stage (A) and show defective accumulation of ß-catenin in mature furrows at the 8-cell stage (C). Embryos from sibling *cei/cei* homozygous mutant females which carry transgenic copies of a maternally-expressed wild-type zebrafish *aurB* gene show significant rescue of the syncytial (B) and ß-catenin furrow accumulation phenotype (D). Side views of 1,000-cell stage live embryos (A,B) and animal views of embryos fixed at the 8-cell stage and labeled with an anti-ß-catenin antibody (green) and a DNA dye (blue) (C,D). Homozygosity for *cei* and presence of the transgene were determined by genotyping as in Materials and Methods.

To confirm that *cei* encodes the zebrafish *aurB* homologue, we attempted to rescue *cei* mutants with a wild-type allele of *aurB*. We used Tol2-mediated transposition [32] to generate transgenic fish carrying the wild-type zebrafish *aurB* cDNA under the control of the *Xenopus* EF-1α promoter, which results in constitutive expression, including during oogenesis ([33],[34]; see Materials and Methods). Non-mosaic transgenic *cei* mutant females were identified by genotyping as described in the Materials and Methods and tested for cellularization defects in their progeny. The presence of the *Tol-2/EF-1α-aurB* transgene resulted in a significant rescue of the *cei* maternal-effect cellularization phenotype (Figure 5, Table 2). For example, while sibling *cei* mutant females lacking the transgene never produced wild-type embryos, normally cellularized embryos are observed in clutches from 9 out of 15 females identified as carrying the *Tol-2/EF1α-aurB* transgene (Figure 5B, compare to Figure 5A, Table 2). Amongst these females, the frequency of rescue to normal cellularization is variable, but can be as high as 92% (Table 2, tg+#1). The variable penetrance and expressivity of the rescue by the transgene may be due to differences in the number of transgene copies in the genome and/or positional effects resulting in different levels of transgene expression, since expression of transgenic constructs is known to be sensitive to positional effects ([35]; our unpublished data). The function of wild-type *aurB* copies may also be counteracted by the antimorphic nature of the *cei* allele (see below), additionally contributing to the observed variable rescue. Consistent with the rescue of the live phenotype by the transgene, ß-catenin accumulation is also restored at mature furrows of 8-cell stage embryos from *cei* homozygous, *Tol-2/EF1α-aurB* carrier females (Figure 5D, compare to Figure 5C).

**Table 2:** Rescue of the *cei* maternal-effect cellularization defect by transgenic copies of *Tol2-EF1a-aurB*.

Maternal genotype (*cei/cei* back.)^1^	Cellularization phenotype (%)^2^ (least severe) (most severe)					n
	normal	c1	c2	c3	c4	
tg(−) #1	0	0	0	8	92	273
tg(−) #2	0	4	6	28	62	47
tg(−) #3	0	0.6	2	11	86	167
tg(−) #4	0	0	0.9	35	64	215
tg(−) #5	0	0.4	0.4	6	93	226
tg(−) #6	0	0	0	4	96	235
tg(−) #7	0	0	0	0	100	63
tg(−) #8	0	0	0	0	100	288
tg(−) #9	0	0	0	0	100	276
tg(−) #10	0	0	0	0	100	232
tg(−) #11	0	0	0.4	54	46	226
total tg(−)	0	0.2	0.4	12	87	2248
tg(+) #1	92	8	0	0	0	131
tg(+) #2	6	14	15	39	27	148
tg(+) #3	3	14	17	40	27	111
tg(+) #4	0	5	24	24	46	37
tg(+) #5	0	0	0	0	100	121
tg(+) #6	15	35	15	8	27	26
tg(+) #7	4	8	14	39	35	158
tg(+) #8	4	5	19	67	6	109
tg(+) #9	0	0	0	0	100	266
tg(+) #10	0	0	0	0	100	88
tg(+) #11	79	20	0.7	0.7	0	135
tg(+) #12	45	21	11	9	14	141
tg(+) #13	0	4	16	38	42	98
tg(+) #14	0.4	2	3	14	81	288
tg(+) #15	0	0.4	2	30	69	273
total tg(+)	15	7	7	20	52	2120

1 Genotype of the females generating the clutch: absence or presence of the *Tol2-EF1α-aurB* transgene (tg(−) or tg(+), respectively) in a *cei/cei* homozygous mutant background. Numbered rows indicate results from individual sibling adult females, derived from four independent founder males, which have been genotyped for the presence or absence of the transgene according to Materials and Methods. Results from the two categories of females have been pooled in the rows labeled “total tg(−)” and “total tg(+)” (highlighted in gray).

2 Fraction of embryos exhibiting cellularization defects of various severities at the 1,000-cell stage, according to the classification presented in Figure 1O–R. For clarity, percentages have been rounded off to the nearest digit, with the exception of values under 1.0, which have been rounded off to the nearest decimal.

### Late Zygotic Function of *cellular island/aurora B* and Preferential Effect of *cei* Maternal-Effect Allele on Early Development

**Figure 6:**
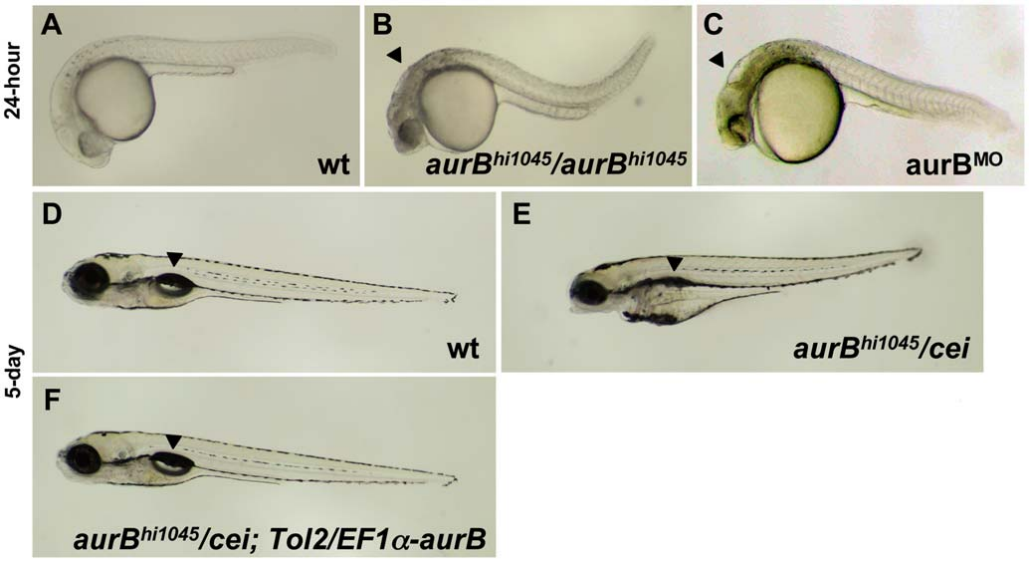
Zygotic *cei/aurB* function is essential for embryonic development. (A–C) Side views of live 24-hour wild-type (A), *hi1045* homozygous (B) and aurB morphant (C) embryos. Arrowheads in (B,C) indicate cell necrosis in the brain. (D–F) Side views of live 5-day wild-type (D) and *hi1045/cei* transheterozygous (E) embryos, as well as *hi1045/cei* transheterozygotes containing transgenic copies of the wild-type zebrafish *aurB* gene (F). Arrowheads indicate the swim bladder, which is inflated in viable embryos (D,F) and indicates inviability when not inflated (E).

**Figure 7:**
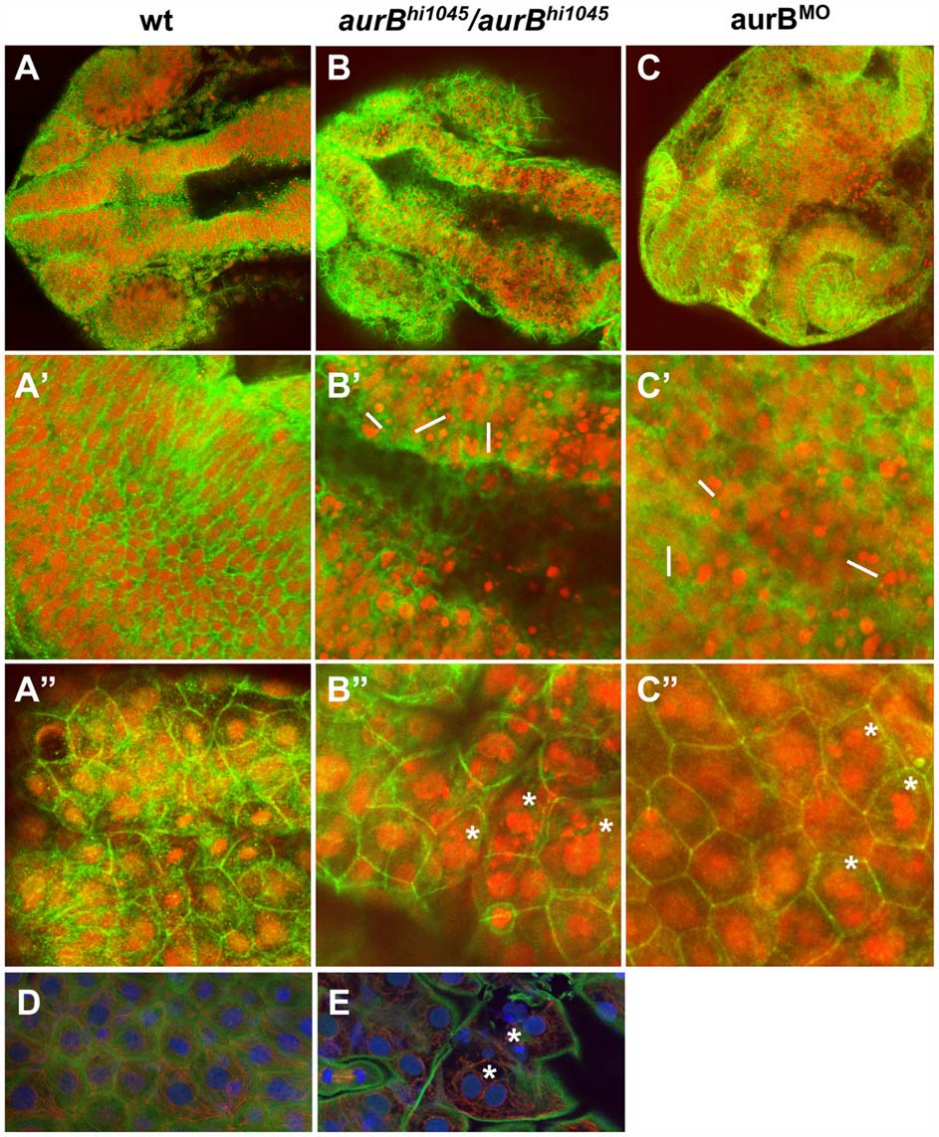
Cytokinesis defects in embryos lacking zygotic *aurB* function. (A–C″) Wild-type (A), *aurB^hi1045^* homozygote (B) and aurB morphant (C) embryos fixed at 24 hours p.f. and labeled to detect ß-catenin (green) and DNA (red). (A–C) Overview of the head region. (A′–C′) Optical section through the brain region, showing internal cells. *aurB^hi1045^* homozygotes and aurB morphants show a high frequency of compact nuclei which are typically arranged in pairs (some examples indicated with a white bar at their left flank), consistent with cell death after a failure to undergo proper cytokinesis. (A″–C″) Optical section through a surface layer of the same region, corresponding to the EVL or peridermal layer. In this layer, cells in *aurB^hi1045^* homozygotes and aurB morphants show a high frequency of multilobular nuclei (some examples indicated by asterisks), again suggestive of defects in cytokinesis. Wild-type embryos injected with control MO exhibit normal cellular and nuclear morphologies (not shown), similar to those observed in untreated wild-type embryos (A). (D–E) High magnification images of a wild-type (D) and *aurB^hi1045^* homozygous (E) embryos labeled to detect ß-catenin (green), microtubules (red) and DNA (blue). Mutant embryos exhibit closely apposed pairs of nuclei (asterisks) that lack an intervening adhesive membrane.

Previous studies had identified two retroviral insertional mutations near the zebrafish *aurora B kinase* gene [36]. Homozygotes for both of these insertions show a brain necrosis phenotype detectable as early as 24 hours p.f. ([36]; Figure 6B, compare to Figure 6A). We tested one of these mutations, originally named *hi1045*, and determined by genomic sequence analysis that the retroviral insertion results in a premature stop codon that leads to the truncation of most of the kinase domain of the protein (Figure S2). Thus, this mutation is a likely null allele of *aurora B kinase*, which we denote *aurB^hi1045^*. We also confirmed that a reduction in zygotic *aurB* function by antisense morpholino-conjugated oligonucleotide (MO)-mediated knock down [37] results in brain necrosis defects similar to those observed in embryos homozygous for the insertional mutations (Figure 6C). To investigate if the brain necrosis phenotype in *aurB^hi1045^* homozygote and *aurB* morphant embryos was caused by defects in cytokinesis, we labeled these embryos to detect nuclei and cell membranes (Figure 7). This analysis showed that, while cells in the developing wild-type brain appear organized in a regular lattice (Figure 7A′) cells in the developing brains of *aurB^hi1045^* homozygotes and *aurB* morphants show regions lacking cellular membranes and which contain high numbers of compact and brightly staining nuclei, which are typically clustered in pairs (Figure 7B′ and 7C′). Embryos with reduced *aurB* function also show defects in the enveloping layer, where a significant number of cells were binucleated or contained multilobular nuclei (Figure 7B″ and 7C″, compare to Figure 7A″). *aurB^hi1045^* homozygous mutant embryos also can show nuclei in close apposition which appear to lack an intervening cell membrane (Figure 7E, compare to Figure 7D), presumably daughter cells which have failed cytokinesis. These observations are consistent with the idea that embryos lacking zygotic *aurB* function exhibit defects in cell division during later stages of embryogenesis, and with a similar function for maternal *aurB* in the pre-midblastula embryo.

The pattern and morphology of nuclei in embryos lacking zygotic *aurB* function suggested that, in these embryos, cells that have failed proper cytokinesis undergo cell death. We confirmed this by labeling live embryos with the apoptosis marker dye acridine orange [38]. *aurB^hi1045^* homozygotes show an increase in acridine orange labeling throughout their body, particularly in the brain region (Figure S3B, compare to Figure S3A). We also tested whether inhibition of caspase activity, which has been shown to be required for cell apoptosis in zebrafish embryos [39], could reduce the brain necrosis phenotype of *aurB^hi1045^* homozygotes. Exposure to the general caspase inhibitor Boc-Asp(OMe)-fluoromethyl ketone (Boc-D-FMK; [40]), results in a significant alleviation of the brain necrosis phenotype in *aurB^hi1045^* homozygous embryos, as well as a reversal of the cell apoptosis phenotype as revealed by acridine orange labeling (Figure S3D, Figure S3D′, compare to Figure S3C, Figure S3C′). Our data indicate that cells that fail to divide due to lack of *cei/aurB* function subsequently undergo cell apoptosis.

To provide additional evidence that *cei* encodes *aurB*, and to further probe the genetic nature of the *cei* allele, we tested the *cei* maternal-effect allele and the *aurB^hi1045^* insertional allele in genetic complementation assays. Embryos from crosses between parents carrying these two mutations appear normal at 24 hours p.f. and do not show the overt signs of brain necrosis observed in *aurB^hi1045^* homozygous and morphant embryos. However, at day 5 p.f., such intercrosses result in inviable embryos in Mendelian proportions consistent with the lethality of *cei/aurB^hi1045^* transheterozygotes (Table 3). These inviable embryos showed defects such as a lack of swim bladder inflation, a protruding mouth and edema in the yolk region (Figure 6E, compare to Figure 6D). Genotyping of individual embryos showed that *cei/aurB^hi1045^* transheterozygotes are inviable at 5 days p.f. in almost all cases (Table 3). Thus, the *cei* maternal-effect allele and the *aurB* insertional mutation fail to complement each other, as expected if they affect the same genetic locus. Introduction of the *Tol-2/EF1α-aurB* transgene rescued the late zygotic lethality associated with *cei/aurB^hi1045^* transheterozygotes (Figure 6F, Table 3), confirming that this lethality is caused by insufficient zygotic *aurB* function. The phenotypes exhibited by *cei/aurB^hi1045^* transheterozygotes are similar to those often caused by late embryonic lethal mutations [41] or exposure to toxic agents (e.g. [42]), suggesting that they may constitute a common phenotypic endpoint for defects in a variety of late developmental pathways. The precise nature of the underlying cause of the lethality of *cei/aurB^hi1045^* transheterozygotes remains to be investigated.

**Table 3:** Transheterozygotes between the *cei* maternal-effect allele and the *aurB^hi1045^* insertional allele are late embryonic lethal, and this lethality is rescued by the *Tol2-EF1α-aurB* transgene.

Cross 1: *cei/+* male×*aurB^hi1045^/*+ female						
day 5 p.f. phenotype^1^	Genotypic classes (%)^2^^,^ 3				total % in phenotypic category	n
	+/+	*aurB^hi1045^/*+	*cei/*+	*cei/aurB^hi1045^*		
Wild-type	25	24	23	1	73	135
Inviable	0	0.7	0	26	27	

Cross 2: *cei/cei* male×*aurB^hi1045^/*+ female				
day 5 p.f. phenotype^1^	Genotypic classes (%)^2^		total % in phenotypic category^3^	n
	*cei/*+	*cei/aurB^hi1045^*		
Wild-type	54	0	54	83
Inviable	0	46	46	

Cross 3: *cei/cei*; *Tol-2/EF1α-aurB* male×*aurB^hi1045^/*+ female						
day 5 p.f. phenotype^1^	Genotypic classes (%)^2^^,^ 3 ^,^ 4				total % in phenotypic category	n
	*cei/+*; *Tol-2/EF1a-aurB*	*cei/+*	*cei/aurB^hi1045^*; *Tol-2/EF1a-aurB*	*cei/aurB^hi1045^*		
Wild-type	26.3	22.2	29.1	1.4	79	162
Inviable	0	1.2	0	19.8	21	

1 Embryonic phenotype on day 5 p.f., classified as either wild-type (normal morphology and swim bladder inflation) or inviable (protruding mouth, edema in the yolk region, darkening of the brain and lack of swim bladder inflation).

2 Genotypic classes of the embryos, as determined by genotyping of individual embryos after phenotypic scoring on day 5 p.f.

3 The presence of rare viable “escaper” *cei/aurB^hi1045^* embryos in cross 1 and *cei/aurB^hi1045^* embryos with no transgene in cross 3 is attributed to residual wild-type zygotic function provided by the *cei* mutant allele (see text).

4 In cross 3, a subset of the phenotypically normal embryos (57/128) were genotyped. The genotypic classes percentage values corresponding to the “wild-type” phenotypic category have been extrapolated from this tested sample.

The observations that the phenotype in *cei/aurB^hi1045^* transheterozygotes is not as severe as that present in embryos homozygous for the *aurB* insertional alleles, and that *cei/cei* homozygotes are viable, suggested that the *cei* allele retains some wild-type function that contributes to zygotic development. We further tested this possibility by expression of Cei/AurB products in *aurB^hi1045^* homozygotes through mRNA injection at the one-cell stage. Expression of products corresponding to either the wild-type or the maternal-effect *cei* alleles significantly alleviates the brain necrosis defects of *aurB^hi1045^* homozygotes (Figure S4B, Figure S4C; compare to Figure S3A). In contrast, injection of mRNA coding for a mutated zebrafish *cei/aurB* product engineered to mimic a known kinase-dead, dominant negative AurB protein (AurBK-R; [43]) results in a brain necrosis defect stronger than that observed in *aurB^hi1045^* homozygotes (Figure S4D). Together with the complementation analysis, these results suggest that the maternal-effect *cei/aurB* allele encodes a partial loss-of-function product that can support cell division at late stages of development.

In spite of the partial function retained by the *cei* maternal-effect allele, heterozygosity for this allele, but not for the *aurB* insertional (null) alleles, results in a dominant maternal-effect phenotype (Table 1). This suggests that under some circumstances the *cei* allele has defects that are more severe than those of a loss-of-function allele. It is possible that the maternal-effect *cei* allele exhibits a complex genetic behavior, acting as a dominant-negative (antimorphic) allele with respect to its maternal function, involved in early embryonic cytokinesis, and as a partial loss-of-function (hypomorphic) allele with respect to its function at later stages of embryonic development (see Discussion).

### Small Molecule Inhibition of AurB Function Mimics the *cei* Mutant Phenotype

Further confirmation of the role of Cei/AurB in furrow initiation was obtained by exposure to the AurB inhibitor ZM2 [44]. Wild-type embryos treated with ZM2 during early development failed to undergo furrow contraction, forming instead rudimentary indentations similar to those observed in embryos from females homozygous for the *cei* mutation (Figure S5). The FMA showed a similar pattern of defects in ZM2-treated wild-type embryos and untreated *cei* mutants, specifically the short, centrally located furrows (Figure S5B″). In addition, a fraction of ZM2-treated embryos exhibited a complete absence of furrowing (Figure S5B′), although we are unable to distinguish whether these defects represent a delay in furrow initiation or a complete failure in furrow formation. Regardless of this uncertainty, these observations are consistent with a requirement of AurB for furrow induction. This pharmacological evidence provides further support for the idea that *cei/aurB* functions in furrow initiation in the zebrafish embryo. As in the case of *cei* mutant embryos, ZM2-treated embryos showed DNA bridges between daughter nuclei (Figure 2D), indicative of DNA segregation defects.

### Expression of *cei/aurB* mRNA in Early Zebrafish Embryos

In situ hybridization showed the presence of uniformly distributed *cei/aurB* mRNA in the early embryo prior to the activation of the zygotic genome at the mid-blastula transition (512- to 1000-cell stage; [45],[46]), indicative of its maternal origin (Figure S6A, compare to labeling with sense probe in Figure S6B). Expression of *cei/aurB* is reduced immediately prior to gastrulation (Figure S6C), presumably reflecting degradation of maternal mRNA and a lower level of zygotic transcription. During gastrulation (Figure S6D) and at the tail-bud stage (Figure S6E), the relative levels of *cei/aurB* expression appear to correlate with an increase in cell number, suggesting that zygotic *cei/aurB* expression is ubiquitous. In the 24-hour embryo, significantly increased expression can be detected in the central nervous system (Figure S6F). This may reflect a higher functional requirement for *aur B* function in this region due to a higher rate of cell division [47], and is consistent with the brain necrosis phenotype caused by the reduction of zygotic *aurB* function. Thus, *cei/aurB* mRNA expression appears to be largely ubiquitous, reflecting an initial large supply of maternal transcript which, when depleted, is substituted by lower levels of zygotic transcript to support ongoing cell division during embryonic development.

### Subcellular Localization of Cei/AurB Protein

**Figure 8:**
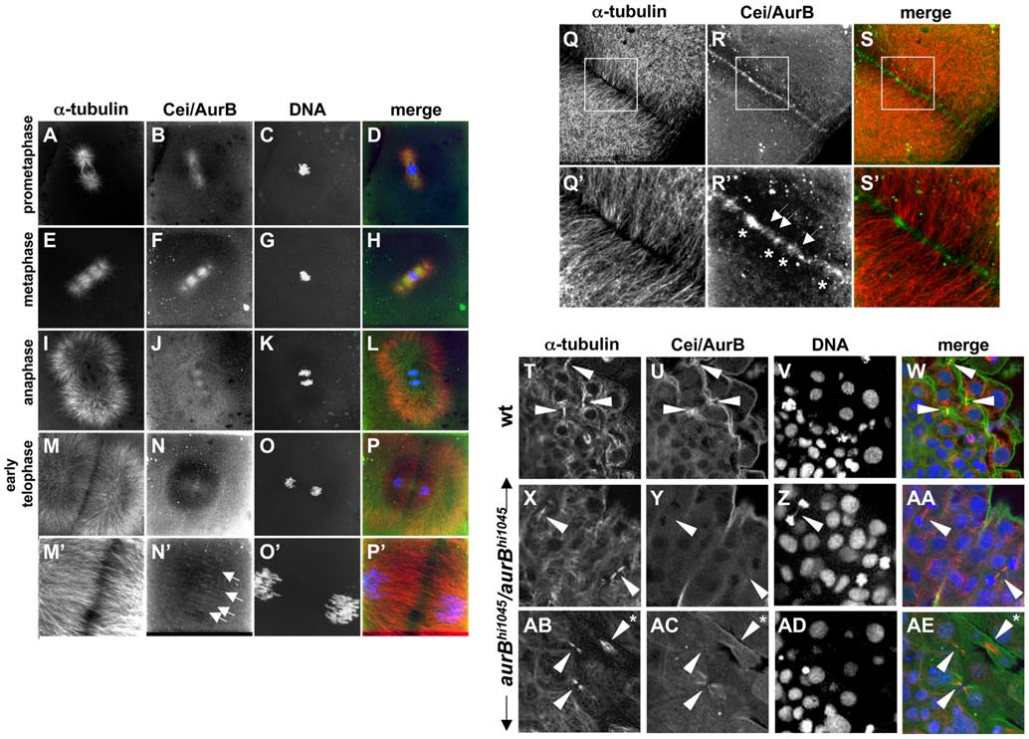
Subcellular localization of Cei/AurB protein. (A–S) Localization of Cei/AurB protein in the early embryo. Animal views of fixed wild-type embryos labeled to detect microtubules, Cei/AurB protein and DNA. (A–P) Series of images detailing the progression of the spindle during the cell cycle as indicated. During prometaphase and metaphase, Cei/AurB protein colocalizes with the forming asters (A–D) and spindles (E–H). During anaphase (I–L), Cei protein remains localized to astral microtubules and the spindle, although localization to the latter appears to subside. During telophase (M–P), Cei/AurB protein transitions to the furrow proper, where it begins to form a ladder-like pattern of short filaments perpendicular to the plane of the furrow (arrows in N′). Panels M′–P′ are 3× magnifications of M–P. (Q–S) During furrow maturation in cytokinesis (shown in this embryo during completion of the first cell division cycle at 38 min p.f.), Cei protein further accumulates along the length of the furrow as punctate aggregates (asterisks in R′) or short filaments perpendicular to the furrow (arrows in R′). These accumulations correspond to sites of microtubule bundling (compare to Q′,S′). Panels Q′–S′ are 3× magnifications of Q–S. All described sites of Cei localization are absent in control labelings using preimmune serum (not shown). (T–AE) Colocalization of Cei/AurB protein to midbodies in fixed 15-somite (16 hours p.f.) embryos. (T–W) Field of cells in a wild-type embryo, showing midbody-like structures (arrowheads in T,U,W). (X–AA, AB–AE) Fields of cells in *hi1045* homozygous mutant embryos. Midbody-like structures are present (arrowheads in X,Y,AA,AB,AC,AE) but exhibit reduced levels of Cei/AurB colocalization and often exhibit a splayed, less compact structure (asterisk in AB,AE). In some cases, DNA bridges can also be observed (arrowhead in Z).

To visualize the subcellular location of Cei/AurB protein in the embryo, we generated an antibody against an N-terminal region of the protein, which differs in length and sequence amongst the various aurora kinase family members (reviewed in [48]). Immunofluorescence analysis shows that Cei/AurB protein localizes to the forming asters and spindles during prometaphase and metaphase (Figure 8A–8H). Astral microtubule and spindle localization remains detectable during anaphase (Figure 8I–8L), although at this stage there appears to be a reduction of Cei/AurB protein at the spindle. During telophase (Figure 8M–8P), coincident with furrow initiation and FMA formation, Cei/AurB protein begins to accumulate at the furrow, where it forms short filaments arranged perpendicular to the plane of the furrow (arrows in Figure 8N′). As the furrow undergoes maturation during cytokinesis (Figure 8Q–8S), Cei/AurB can be observed in a punctate pattern along the plane of the furrow. Upon closer examination, this punctate pattern represents a collection of short filaments, as before arranged perpendicular to the furrow but now with an apparent greater labeling intensity (arrows in Figure 8R′), as well as larger aggregates (asterisks in Figure 8R′). Because FMA tubules are thought to be derived from astral microtubules (compare Figure 8I and 8M), this localization pattern is consistent with an association of the Cei/AurB protein with plus ends of astral microtubules, as described in other systems [7],[8]. Importantly, a comparison with the microtubule localization pattern indicates that the sites of Cei protein localization correspond to sites of greater microtubule bundling (Figure 8Q′–8S′). Moreover, the intensity and size of the Cei/AurB signals appears to correlate with the apparent number of microtubules undergoing bundling. Levels of furrow associated Cei/AurB become reduced at later stages of cytokinesis, when furrows are nearing their completion (data not shown). These observations are consistent with a role for AurB in furrow initiation and maturation.

The zygotic lethality associated with the *cei/aurB* null phenotype indicates a role for zygotically derived Cei/AurB protein in later stages of development. Therefore, we also analyzed Cei/AurB localization in embryos at the 15 somite stage (16 hours p.f.; Figure 8T–8AE). We analyzed embryos derived from heterozygotes of the presumptive null insertional *aurB* mutation *aurB^hi1045^*, such that the 25% of the progeny corresponding to the zygotically homozygous *aurB^hi1045^* (*aurB^hi1045^*/*aurB^hi1045^*) mutants could act as a control for the specificity of the antibody. As mentioned above (see also Figure 7), homozygous *aurB^hi1045^* mutant embryos typically exhibit abnormal DNA morphology, such as highly condensed nuclei characteristic of apoptotic cells, which allow them to be distinguished from wild-type (*+/+* or *aurB^hi1045^*/*+*) embryos. Colabeling with an anti-α-tubulin antibody allowed visualization of the microtubule apparatus. In wild-type embryos, Cei/AurB protein localized to microtubule-based structures reminiscent of midbodies which were present at regions of cell-cell contact between presumed daughter cells at telophase (arrowheads in Figure 8T, 8U, and 8W). Such structures were still observed in *aurB^hi1045^* homozygotes, although the levels of Cei/AurB were either strongly reduced or absent (arrowheads in Figure 8X, 8Y, 8AA; Figure 8AB, 8AC, 8AE). Intriguingly, although in wild-type embryos these midbody-like structures are highly compact (arrowheads in Figure 8T), in homozygous mutant embryos a significant fraction of midbody-like structures were splayed in a noticeably less compact arrangement (asterisk in Figure 8AB): 33% midbodies had a splayed morphology in mutants (n = 75 midbodies, analyzed in 8 embryos), compared to 3.5% in sibling embryos (n = 121, analyzed in 16 embryos). These experiments suggest that zygotically-derived Cei/AurB protein specifically localizes to the midbody in cleaving cells of the later embryo and are consistent with a zygotic function of this protein in cytokinesis and midbody organization. As in early embryos with reduced AurB function, DNA bridges can also be observed in zygotically mutant embryos (arrowhead in Figure 8Z), indicating a role for zygotic Cei/AurB function in DNA segregation in the later embryo.

We also determined the localization of Cei/AurB protein in maternally mutant *cei* embryos. In these embryos, Cei/AurB protein exhibits an apparently normal astral and spindle localization (data not shown) and accumulates, as in wild-type embryos, at the ends of FMA tubules in the shortened furrow (Figure S7A–S7C). Thus, the shortened furrows that form under conditions of reduced *cei* function are able to recruit Cei protein to its normal site of localization. This experiment also shows that, in spite of its presumed dominant negative character, the maternally mutant Cei/AurB product undergoes an apparently normal subcellular localization.

### Role of Maternal *cei/aurB* Function in Germ Plasm Furrow Recruitment

**Figure 9:**
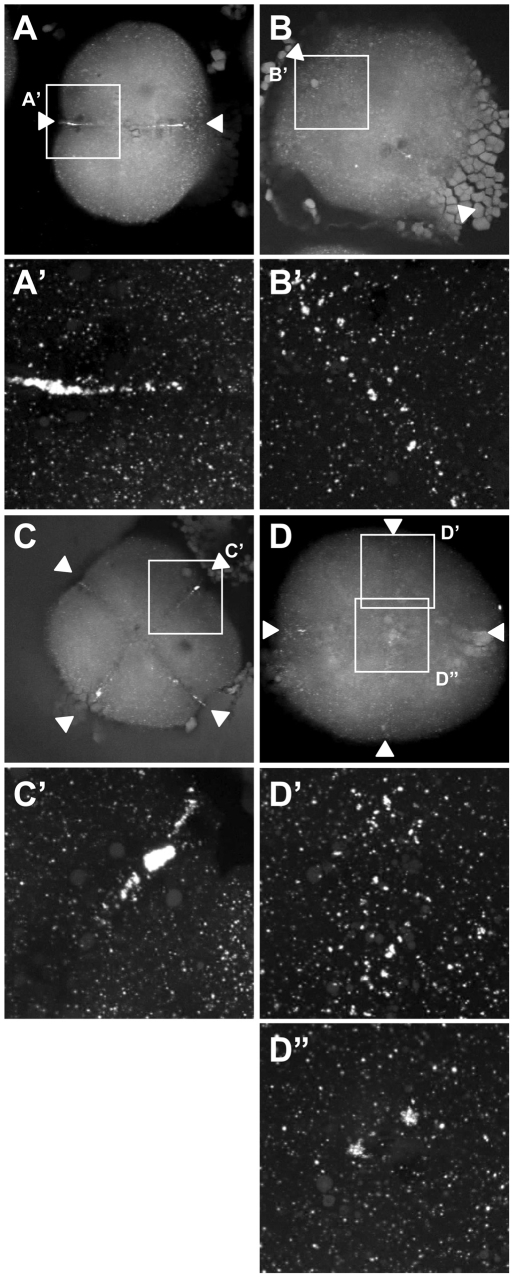
Germ plasm recruitment in *cei/aurB* mutant embryos. Localization of *vasa* mRNA by fluorescent in situ hybridization in wild-type embryos fixed at 49 min p.f. (A,B) and 65 min p.f. (C,D). In the wild-type, germ plasm becomes recruited as an elongated rod-like structure in the approximately 2/3 distal regions of the furrow (A′), then localizes as a compact structure at the distal furrow end (C′). Recruitment of germ plasm is reduced or absent in *cei* mutants (B′, D′). Instead, aggregated germ plasm often becomes recruited to two aggregates in the center of the blastodisc, corresponding to the edges of the truncated furrow-like structure observed in these mutants (D″). Arrowheads flank planes of cleavage.

Previous studies suggest that germ plasm granules present in the zebrafish oocyte cortex undergo a process of aggregation during the first cell cycle, dependent on the alignment of a cytoskeletal network to which they are bound [23]. In a wild-type embryo, aggregation occurs concomitant with furrow formation, such that a collection of larger aggregates, composed of collected smaller granules, becomes recruited to the forming furrow in an elongated rod-like structure (Figure 9A and 9A′). Because the cortex of the wild-type fertilized zygote contains a granule-free zone at its animal-most region, germ plasm recruitment occurs in the approximately 2/3 distal-most regions of the blastodisc ([23]; Figure 9A and 9A′). This rod-like structure later undergoes further aggregation as it forms a compact mass at the distal end of the maturing furrow ([13],[17],[23],[49]; Figure 9C and 9C′).

Because the distal furrow region where the germ plasm normally becomes recruited is precisely the region most sensitive to the loss of *cei/aurB* function, we expected to observe defects in germ plasm recruitment in *cei/aurB* mutant embryos. To detect germ plasm recruitment in the early zebrafish, we carried out fluorescent in situ hybridization labeling of wild-type and *cei/aurB* mutant embryos to detect *vasa* mRNA, a component of the zebrafish germ plasm (Figure 9). This technique allows detecting individual cortical germ plasm granules as they aggregate during the first cell cycle ([23] – similar results, albeit with a reduced resolution, have been obtained using the standard color substrates, data not shown). In distal regions of the furrow, *cei/aurB* mutant embryos exhibited a reduction in germ plasm recruitment, often showing partially aggregated germ plasm granules that do not form well-defined rod-like structures (Figure 9B, 9B′, 9D, and 9D′). Interestingly, germ plasm granules were often seen recruited as pairs of large aggregates in medial regions of the furrow and flanking the shortened furrow-like structure that forms in these mutants (Figure 9D″ and data not shown). These experiments indicate that germ plasm recruitment depends on cei/aurB function, possibly because cei/aurB function is important for the organization of the cytoskeleton to provide a stable germ plasm anchoring structure. Moreover, this anchoring structure appears to be present in the shortened medial furrows that do form in *cei/aurB* mutants.

### The Medial Furrow Signal Remaining in *cei* Mutants Depends on the Mitotic Spindle

The presence of medially located, shortened furrows in *cei* mutant embryos was intriguing, especially because such structures appeared otherwise relatively normal. This was particularly evident in the case of the FMA, where the length of the recruited microtubules themselves is similar to that of FMA tubules in wild-type embryos. Moreover, in *cei* mutants the length of FMA tubules is similar along the span of the truncated furrow, arguing that a sharp functional threshold may exist above which furrow structures form normally (in medial regions) and below which furrow structures do not develop (in distal regions). We wondered why a shortened furrow forms in the medial region of the blastodisc in *cei* mutant embryos. Given previous work in other systems indicating redundant astral- and mitotic spindle-derived signals in furrow initiation [3],[7],[8], one possible explanation for the shortened, medially located furrow is induction by the mitotic spindle in the absence of astral, *cei*-dependent signals. An alternative explanation is that astral microtubule density is higher in the medial region due to aster overlap, which results in an above-threshold AurB activity allowing furrow induction in medial regions. In order to discern between these two possibilities, we asked whether the shortened furrow that develops in *cei* mutants depends on a mitotic spindle-derived signal. For this purpose, we took advantage of another zebrafish maternal mutant, of the gene *futile cycle (fue)*, which lacks mitotic spindles (in addition to having pronuclear fusion defects; [31]). In spite of these defects, *fue* mutant embryos undergo a relatively normal cell cleavage pattern, presumably driven by the normal cycle of centrosome duplication and aster formation [31].

**Figure 10:**
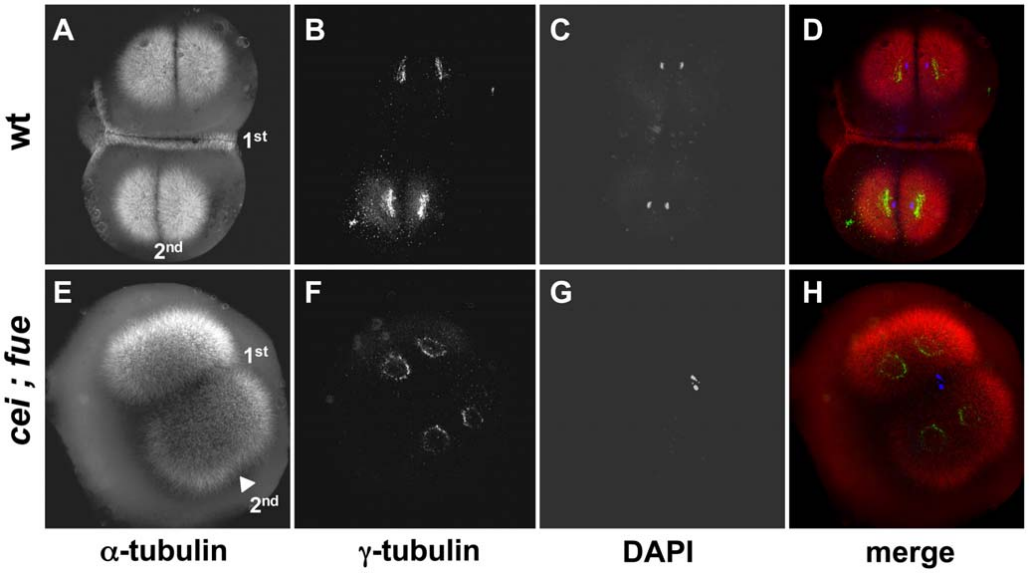
Redundant signals contribute to furrow formation in the early blastomeres. Wild-type (A–D) and *cei*; *fue* double mutant (E–H) embryos were fixed at 49 min p.f. and labeled with anti–α-tubulin antibodies (A,E; red in D,H), anti–γ-tubulin antibodies (B,F; green in D,H) and DAPI (C,G; blue in D,H). (A–D) At this stage, wild-type embryos exhibit an FMA along the span of the first cleavage plane (1^st^). (E–H) *cei*; *fue* double mutant embryos lack an FMA in either medial or distal regions of the 1^st^ cleavage plane (compare to Figure 3D,H). Double mutants show the expected delay in furrow induction, characteristic of the *cei* mutation, in the less mature furrow corresponding to the 2^nd^ cleavage plane (arrowhead in (E); compare to Figure 3B and 3F). Double mutants also exhibit the pronuclear fusion defect characteristic of the *fue* mutation [31]. Similar results can be observed in embryos until 65 min p.f. (not shown).

Females maternally homozygous for both *cei* and *fue* mutations were generated through genetic crosses and identified through genotyping of flanking linked polymorphic DNA markers. Embryos from these females, produced through crosses to wild-type males, did not show any sign of furrow formation (not shown). Because such embryos could not be distinguished from normal, unfertilized eggs, we labeled fixed samples to detect sperm-derived structures such as the male pronucleus and the sperm-dependent centrosome. Fixed double mutant embryos were labeled to detect pronuclei with a DNA stain, centrosomes with an anti-γ-tubulin antibody, and microtubules with an anti-α-tubulin antibody (Figure 10). Imaging of double mutant embryos revealed the presence of two pronuclei and dividing centrosomes, demonstrating that these embryos had been fertilized and were undergoing centrosomal duplication cycles (Figure 10F and 10G). However, in spite of a normal centrosome duplication pattern, furrow associated structures, such as the FMA, were completely lacking in double mutant embryos (Figure 10E, compare to Figure 10A). These results suggest that the shortened furrows observed in *cei* mutant embryos are induced by a spindle-derived signal.

Double labeling experiments show that, in *fue* single mutant embryos, both FMA formation and Cei/AurB protein localization to the furrow appear normal and occur throughout the span of the blastodisc (Figure S7D–S7F), and that Cei/AurB protein localizes to the forming asters at earlier stages of the cell cycle (data not shown). This is consistent with Cei/AurB protein being solely provided by astral microtubules in these mutant embryos, which lack a spindle apparatus.

Together, our results provide further support for models where furrow induction depends on redundant signals derived from the aster and spindle microtubules. Moreover, our data indicate that in large cells such as in the early zebrafish embryo, these redundant structures may have distinct roles in medial vs. distal regions of the cell. Specifically, the maternal-effect *cei* mutation affects furrow initiation in distal region of the blastodisc, while furrow initiation in medial regions may depend on redundant astral and midzone-derived signals.

## Discussion

We show that zebrafish *cellular island* encodes Aurora B kinase, and is essential for furrow initiation and germ plasm recruitment in the early embryo. Positional cloning, sequence analysis, genetic rescue and complementation analysis indicate that the effects on cytokinesis caused by the *cellular island* maternal-effect mutation result from an amino acid change in an evolutionarily conserved residue at a position near the activation domain essential for Aur B kinase activity. This report constitutes one of the first examples of a thorough molecular and functional analysis of a vertebrate maternal-effect gene after its identification through forward genetics [24],[25],[27],[28], demonstrating the validity of this unbiased approach. It also constitutes a unique case of a genetic mutation in a vertebrate gene dedicated to the process of cytokinesis. This analysis has provided a number of novel insights into the function of Aurora B kinase in the early vertebrate embryo, such as distinct requirements for induction in medial and distal regions of the blastodisc and a role in the reorganization of the cytoskeleton during furrow maturation. The complex genetic nature of the *cei* mutation also suggests the presence of different mechanisms of cytokinesis in early and late embryos.

### Multiple Furrow-Inducing Signals Have Differing Roles in Medial and Distal Regions of the Blastodisc

**Figure 11:**
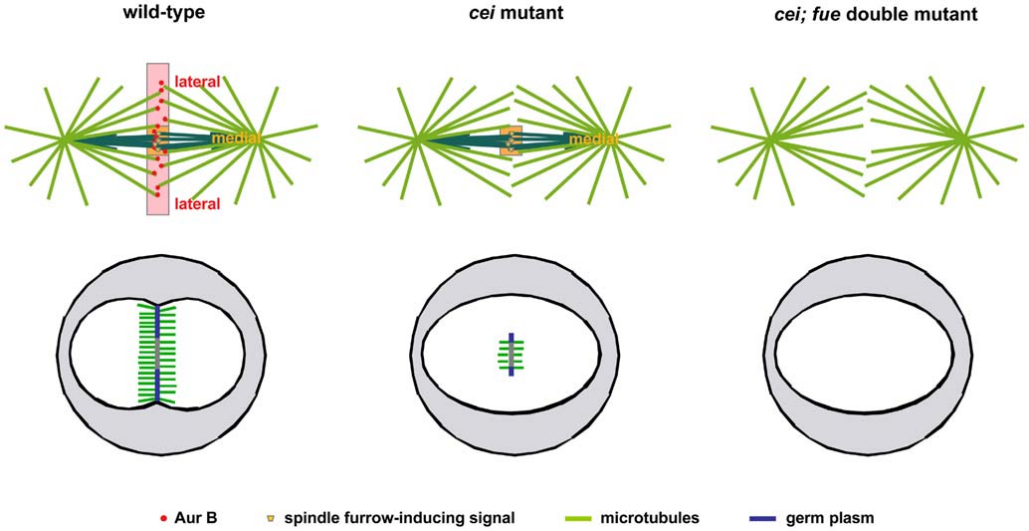
Model for the action of *cei/aurB* and other furrow induction signals in the early zebrafish embryo. Top images: Cei/AurB protein associated with astral microtubule ends and midzone-derived signals constitute partially redundant signals for furrow formation in the early zygote. Both astral microtubule- and spindle-derived signals act redundantly to initiate furrow formation in the medial region of the early embryonic blastodisc. On the other hand, astral-derived signals, dependent on *cei* function, are essential for furrow formation in distal regions of the blastodisc. Our analysis does not rule out a role for Cei/AurB, which is also localized to the spindle, as a mediator of a spindle-derived signal. Bottom images: Furrow induction results in the formation of furrow-associated cytoskeletal structures such as the FMA (green), as well as germ plasm recruitment (blue).

It is remarkable that embryos from *cei* mutant females typically display a shortened furrow-like structure in the medial region of the blastodisc, but a complete lack of furrowing in more distal regions. Interestingly, Cei/AurB protein is found throughout the length of the furrow of the early embryo, and exposure to the AurB inhibitor ZM2 can result in a complete absence of furrow induction. Thus, our observations suggest that AurB function is important throughout the length of the furrow, but that the medial region of the furrow is more resilient to a partial loss of AurB activity (as in the case of the *cei* mutation) than distal regions. Because the region of maximum overlap of astral microtubules is at the center of the forming furrow, one explanation for the spatially limited furrow in *cei* mutants is an increased density of astral microtubule ends, and consequently astral-derived signals, in the center of the blastodisc. An alternative explanation for the spatially restricted furrow induction phenotype is that medial furrow regions, which may be influenced by the centrally located mitotic spindle, are induced by redundant mechanisms mediated by both astral and spindle microtubules. We discriminated between these two possibilities by examining furrow formation in *cei* mutants in a background also mutant for *futile cycle*, which additionally lack mitotic spindles but have normal astral microtubules. In *cei*; *fue* double mutants, the cell cycle proceeds normally, as reflected by the pattern of centrosome duplication, but furrow initiation is completely abolished both in distal and medial regions of the blastodisc. Thus, our data supports previous findings that spindle and astral microtubules act together to induce furrows in the *C. elegans* embryo [3],[4] and in HeLa cells [5], and shows that a similar redundancy occurs in the early zebrafish embryo. Our analysis shows for the first time that these signals are spatially dedicated: the spindle-derived signal(s) having an influence only in medial regions of the blastomere, while astral-derived signal(s) are essential for furrow initiation in more distal regions (Figure 11). It is possible that this dual mechanism arose in order to form a furrow throughout the span of the large blastomeres of the early vertebrate embryo. Spatial differences in the molecular machinery may also be important to generate asymmetries important for embryonic development, for example, in the segregation of localized determinants to specific locations of the zygote. While our results indicate that Cei/AurB protein is essential for the astral-mediated furrow-inducing signal, they do not rule out a role for Cei/AurB as a mediator of a spindle-derived signal. Indeed, both the spindle localization of Cei protein and drug inhibition experiments suggest that this protein may also be at least partially involved in mediating a spindle-derived furrowing activity. Further analysis will be required to determine the precise molecular nature of these two sets of partially redundant signals.

### Evidence Suggesting Differing Mechanisms of Cytokinesis in Early and Late Embryos

The unusual genetic behavior of the maternal-effect *cei* mutation, in comparison to that of null insertional alleles, is intriguing. On one hand, the *cei* mutant allele appears to retain some aspects of wild-type function. For example, the *aurB* null phenotype is zygotic lethality involving brain necrosis detectable at 24 hours p.f., but embryos homozygous for the *cei* mutant allele (derived from *cei/+* heterozygous parents) are viable and can reach adulthood. Similarly, transheterozygotes for the *cei* maternal-effect mutation and null alleles do not exhibit early brain necrosis and survive until day 5 p.f. In addition, expression of the product corresponding to the maternal mutant *cei* allele, as is also the case with the wild-type allele, is able to ameliorate the zygotic brain necrosis defect caused by homozygosity for a null *cei/aurB* allele. Thus, multiple lines of evidence indicate that the *cei* maternal-effect allele retains sufficient wild-type function to partially support cell division during zygotic development.

On the other hand, maternal heterozygosity for the *cei* mutation, but not for the *aurB* insertional alleles, results in a significant dominant effect in cytokinesis in the early zygote. Although we can not rule out the possibility that this contrasting genetic behavior is due to genetic background differences, these results suggest that, with regards to a maternal-effect, the *cei* allele may have a dominant negative character. This apparent dominant negative effect can be mimicked by treatment with a small molecule AurB inhibitor, indicating that the underlying cause of the defect is the inhibition of endogenous AurB function. Together, these data suggest that the mutation in the *cei* maternal-effect allele may specifically interfere with *aurB* function and cytokinesis during the early cleavage divisions while largely retaining normal *aurB* function during later embryonic development. This finding suggests possible mechanistic differences in furrow formation pathways between large blastomeres in the early embryo and smaller cells at later stages. Potential bases for this differential functional requirement may originate on specific constraints for cell division in the early zygote, for example an increased reliance on astral-derived signals for furrow induction in the larger early blastomeres, differences in cell cycle length, or a different architecture of the furrow associated microtubule cytoskeleton (FMA vs. midbody) during furrow maturation. Regardless of potential differences in its function in early and late embryonic development, *cei/aurB* function appears to have a crucial role in cytokinesis in both early and late embryos.

The observation that the first defects seen in zygotic null *aurB* mutants occur in the brain region may reflect the fact that this region contains the most actively dividing cells in the developing embryo. Increased rates of cell division would in turn lead to faster depletion of maternally provided protein supplies, as previously suggested for other general cell cycle genes [47]. One of the manifestations of this phenotype is cell death in the brain region. We show that this cell death is associated with cells typically arranged in pairs and which exhibit DNA morphologies characteristic of apoptotic cells. Our observations in zebrafish are consistent with previous reports that inhibition of *aurB* function results in cell death [44]. The extensive cell death caused by loss of zygotic *aurB* function in these null mutants contrasts with the lack of any such effects after aberrant cytokinesis during the cleavage stages in *cei* mutant embryos. It has been proposed that early zebrafish embryonic cleavage divisions lack cell cycle check points [39],[50],[51], which may explain the ability of the early embryo to continue nuclear division in the absence of cytokinesis without entering the apoptotic program. Thus, cells in early and late embryos may differ not only in their precise mechanisms of cytokinesis but also in the cellular response to failures in these mechanisms.

### Role for AurB Kinase in Cytoskeletal Reorganization during Furrow Maturation

In wild-type zebrafish embryos, furrow formation and maturation is accompanied by striking cytoskeletal rearrangements, involving the reorganization of the actin and microtubule-based cytoskeleton and the formation of cell adhesion junctions [10],[12]. Some events associated with early furrow formation, such as the formation of the FMA, occur normally in the partial furrows observed in *cei* mutants. However, subsequent events associated with furrow maturation do not occur normally in these mutants. The FMA, for example, does not undergo the tilting, distal enrichment and eventual disassembly characteristic of wild-type embryos, and instead remains as a stabilized structure containing parallel microtubules. Two observations suggest that the FMA reorganization defect in *cei* mutants is not simply a consequence of the shortened, medially located furrows. First, this cytoskeletal reorganization defect occurs even in embryos showing a weak effect where the furrow encompasses the entire blastomere. Secondly, FMA tubules can become locally reorganized even in short and medially located stretches of a furrow, as in the case of *nebel* mutant embryos [13]. Thus, our observations are consistent with a role for *cei/aurB* function in cytoskeletal reorganization during furrow maturation. Such a role is also supported by our observations that, during later embryonic development, mid-body like structures in embryos with reduced AurB function often exhibit a splayed phenotype. It is possible that both the lack of FMA reorganization in early blastomeres and the splayed midbody phenotype in later cells are caused by reduced microtubule bundling. In support for this idea, we have found that Cei/AurB protein normally colocalizes in patches and short segments perpendicular to the cleavage plane along the maturing furrow, and that these localization domains coincide with the tips of bundling FMA tubules. The idea that Cei/AurB mediates microtubule bundling is consistent with studies that show a role for the Chromosomal Passenger Complex proteins in spindle formation and microtubule bundling during the initiation of cytokinesis [52]–[55]. To our knowledge, this is the first evidence that suggests a role for AurB in microtubule reorganization at later stages of furrow formation, important for furrow maturation and completion. In this respect, the large cytoskeletal rearrangements present in the early zebrafish embryo may provide a useful system to study this function. AurB is known to regulate the activity of microtubule bundling factors such as Mklp1 [56],[57], and Mklp1 is required for cytokinesis in the early zebrafish embryo [58]. Further research, including live imaging and ultrastructural analysis of the microtubule network, will address and attempt to fully validate a role of zebrafish *cei/aurB* and other factors such as Mklp1 in microtubule bundling.

### *cei/aurB* Function and Germ Plasm Recruitment

Consistent with the lack of distal furrows in *cei* mutant embryos, germ plasm granules exhibit reduced aggregation in these regions. Instead, germ plasm aggregates accumulate in paired structures flanking the medially located shortened FMA. Germ plasm recruitment at the edges of the shortened furrow in *cei/aurB* mutants, but not in more distal regions, suggests that furrow formation signals promote the establishment or stabilization of anchors for germ plasm recruitment (Figure 11). Of interest, the shortened spindle-dependent furrow present in *cei* mutants appears to be as effective as a wild-type, full-span furrow with regards to germ plasm recruitment, suggesting that induction by either spindle- or astral-dependent signals results in similar furrow architectures and/or functions. In addition to a role for *cei/aurB* function in furrow initiation, and therefore germ plasm recruitment, our findings suggest a role for this gene in cytoskeletal rearrangements during furrow maturation, namely the tilting and distal enrichment of FMA tubules. As this FMA reorganization is associated with the translocation of germ plasm to the distal ends of the furrow and its higher-level aggregation in these regions [10], *cei/aurB* function may also be important for this later step in germ plasm segregation.

In summary, we show that a maternal-effect allele in *cellular island* is caused by a mutation in the zebrafish *aurora B kinase* homologue. Functional analysis of this gene indicates that *aurB* is essential for various functions involved in cell division in the early embryo, including furrow formation and germ plasm recruitment, and is also required for cell division during later stages of embryogenesis. We further show that a dual system, involving astral- and spindle-derived signals and mediated at least in part through *cei/aurB* function, ensures furrow formation throughout the length of the large blastomeres of the early embryo. Finally, we provide evidence that supports a role for *aurB* in microtubule reorganization during late stages of cytokinesis.

## Materials and Methods

### Genetic Stocks and Methods

Wild-type stocks were the standard AB. Fish and embryos were raised and maintained under standard conditions at 28.5°C [59]. Fish carrying the *cei* allele (*cei^p63cd^*) were genotyped by using the flanking linked markers z10673 and z51215 or direct sequencing of the PCR fragment of genomic DNA amplified using the following primers: 5′-GCATCCCAACATCCTTCGCTTCTAC-3′ and 5′-AGTAGCAGTGCGCTGATCGTCAAAG-3′ to detect the mutation site. In vitro fertilization was carried out as previously described [24].

Females doubly homozygous for the *cei/aurB* and *fue* mutations were produced by crossing *cei* homozygous males to heterozygous *fue* females. Linked markers were used to identify double heterozygotes from this original cross and these individuals were then mated to produce full homozygous double mutants. Genetic identity of the *cei*; *fue* mutants was confirmed using RFLP markers corresponding to the polymorphic bases responsible for each mutant phenotype.

All animals were handled in strict accordance with good animal practice as defined by the relevant national and/or local animal welfare bodies, and all animal work was approved by the appropriate committee (University of Wisconsin - Madison assurance number A3368-01).

### Positional Cloning of *cellular island*

Males heterozygous or homozygous for the *cei* mutation were crossed to wild-type WIK females to generate F_1_ families, which were incrossed to obtain homozygous F_2_ fish [60]. The early cell cleavage phenotype of F_3_ embryos allowed determination of the genotype of F_2_ females with respect to the *cea* mutation, which was compared to the segregation of flanking SSLP markers. Two fragments of aurB cDNA covering the entire AurB coding region were amplified from one-cell stage *cei* mutant embryos by RT-PCR using the following restriction enzyme recognition sequence attached primer pairs: N-terminal fragment: 5′-ggaattcCGAAACACACACACACACACG-3′and 5′-gggtcgacGTTCTCCTCTGTATCCCAGC-3′; C-terminal fragment: 5′-ggaattcAGAAGGTGATCCACAGAGAC-3′ and 5′-gggtcgacAGGTGTGTGTATATGCCAGG-3′. These fragments were cloned into the EcoR1/Sal1 digested pBluescript SK(+) vector and sequenced. To confirm linkage between the *cei* mutation and the mutation in *aurB*, we amplified a partial sequence of *aurB* containing the mutation region by PCR using the primers described in the Genetics stocks and methods subsection. Sequencing of the *aurB^hi1045^* allele was carried out using genomic DNA from the insertional line as specified in [36].

### Immunofluorescence, In Situ Hybridization, and Imaging

For the detection of tubulins and ß-catenin, dechorionated embryos were fixed and labeled as previously described [23] using microtubule fix buffer and the following primary antibodies: anti-α-tubulin (Sigma, monoclonal B5-1-2, 1∶2500), anti-γ-tubulin (Sigma, monoclonal GTU-88, 1∶2000; Sigma, rabbit polyclonal, 1∶2000), and anti-ß-catenin (Sigma, rabbit polyclonal, 1∶1000). The primary antibodies were recognized using Alexa 488- or Cy3-conjugated secondary antibodies (Molecular Probes, Jackson Immuno Research Laboratories). Staining of f-actin was determined using Alexa-488 phalloidin (Molecular Probes) as in [10]. Subsequently, embryos were labeled for DNA using DAPI (0.5 µg/ml in PBS) for 10 minutes at room temperature or propidium iodide (described in [13]). In situ hybridizations were carried out as described previously [61] using *vasa* [17] or *cei/aurB* RNA probes. The *cei/aurB* antisense probe was generated by the EcoR1 digestion of pBS-N terminal-*aurB* (see Positional cloning subsection) with T7 polymerase.

Anti-Cei/AurB was generated by immunizing rabbits against a KLH-conjugated peptide containing the first 29 amino acids of zebrafish AurB (Harlan Laboratories). Preabsorbed antibody was used on embryos fixed with microtubule fix at a 1∶100 dilution.

An acridine orange (Sigma) stock solution was made at 4 mg/ml in water and dechorionated embryos were exposed to 0.4 mg/ml in embryonic medium for one hour. Embryos were subsequently washed extensively with embryonic medium prior to imaging.

Live and fluorescently labeled single embryos were imaged using a Zeiss Axioplan2 fluorescent microscope and Open Lab imaging software. Images of embryos labeled using in situ hybridization and groups of live embryos were acquired using a Leica-FLIII and a color camera (Diagnostic Instruments Spot Insight). Confocal microscopy was carried out using a Zeiss LSM 510 confocal microscope and the acquired images were processed using Image J software.

### Drug Exposure, Morphant Analysis, and mRNA Injection

ZM2 (ZM44739, Tocris Bioscience) was dissolved in DMSO at a concentration of 20 mM. Dechorionated embryos were exposed to 400 µM ZM2 diluted in embryonic medium starting at 25 min after fertilization until fixation at the indicated stage. The general caspase inhibitor Boc-Asp(OMe)-fluoromethyl ketone (Boc-D-FMK; Sigma) was dissolved in DMSO at a concentration of 20 mM, and dechorionated embryos were exposed to 400 µM Boc-D-FMK in embryonic medium beginning at 17 hours p.f. until indicated. For drug exposure experiments, control embryos were exposed to a similar concentration of carrier solvent (DMSO).

AurB morphant embryos were generated by microinjection at the one-cell stage of 0.5 ng MOs of the following sequence: 5′ CGGTTTTCTTTATTCTGCATGGCG 3′ (GeneTools). For overexpression of wild-type and maternal-effect *cei* allele, cDNAs corresponding to these two alleles were cloned into pCS2. The AurB(K-R) expression vector was generated by substituting in the wild-type allele a lysine (AAG) at amino acid position 82 by an arginine (AGG) through PCR-based directed mutagenesis, to recreate a kinase dead dominant negative mutation [43]. In vitro transcribed mRNA was prepared and injected at a concentration of 0.5 ug/ul as previously described [61].

### Transgenic Production and Genotyping

The full-length wild type cei/aurB cDNA was cloned into the pT2KXIGDin vector under the expression of the EF1α promoter [34]. DNA for the resulting construct, *Tol-2/EF-1α-aurB*, together with Tol2-transposase, was injected into embryos derived from crosses between zygotically *cei* homozygous males and heterozygous females to produce individuals that were carriers for both the mutation and the transgene. In these injections, 25 pg of plasmid DNA was injected into one-cell stage embryos together with 50 pg of in vitro transcribed Tol2 transposase mRNA [62]. Founder individuals carrying the transgene were identified by PCR-based genotyping and were intercrossed to generate homozygous *cei* mutant females that are *Tol-2/EF1α-aurB* non-mosaic carriers. The presence of the transgene in transgenic fish was detected by PCR using following primers: (5′-GAAGAAGGTGATCCACAGAGAC-3′ and 5′-AAACACTCGTAGCACAGCACAC-3′).

## Supporting Information

Figure S1 The *cei* mutation involves the substitution of a conserved amino acid in aurora B kinase. (A) Genetic linkage of the *cei* mutation to a region of Chromosome 14. Linked DNA markers and number of genetic recombinants over total number of analyzed meiotic events are indicated. (B) Sequence comparison of the zebrafish, *Xenopus*, and human Aurora B kinase protein. The red asterisk indicates the position of the amino acid substitution associated with the *cei* mutation. (C) Sequence conservation in the region of the identified amino substitution. The mutation results in a Valine→Methionine substitution at a position that is highly conserved amongst Aurora kinases but not conserved in the less related kinase Plk4.
(5.77 MB TIF)

Figure S2 Retroviral insertion in the *aurB^hi1045^* allele results in the truncation of zebrafish AurB. (A) Structure of the zebrafish *aurB* gene, depicting intron-exon boundaries, the kinase domain of the protein (in green) and the location of the retroviral insertion. (B) Sequence in the boundary region between the N-terminal portion of the AurB protein in the wild-type allele and in the *aurB^hi1045^* allele, showing the premature stop codon caused by the retroviral insertion that results in the truncation of most of the kinase domain of AurB.
(0.84 MB TIF)

Figure S3 *aurB^hi1045^* homozygotes exhibit an increase in cell apoptosis. (A,B) Exposure to the live cell apoptosis dye acridine orange brightly labels *aurB^hi1045^* homozygotes (B), while control siblings only exhibit background levels of labeling (A). (C–D) Embryos from incrosses between *aurB^hi1045^* heterozytes, 25% of which are expected to be *aurB^hi1045^* homozygotes, were treated with either the caspase inhibitor Boc-D-FMK (D,D′) or control solvent (DMSO; C,C′). Embryos exhibiting the brain necrosis phenotype characteristic of *aurB^hi1045^* homozygotes are indicated with an asterisk. Boc-D-FMK treatment prevents both the brain necrosis visible under standard microscopy (DIC; D, compare to C) and the accumulation of acridine orange labeled cells (AO; D′ compare to C′).
(7.77 MB TIF)

Figure S4 Expression of Cei/AurB products in *aurB^hi1045^* homozygotes. mRNA was injected at the one-cell stage into embryos derived from incrosses of *aurB^hi1045^* heterozygotes, so that an expected 25% of the progeny are *aurB^hi1045^* homozygotes. In (A–C,A′–C′), embryos showing overt signs of necrosis are presumed to be *aurB^hi1045^* homozygotes and are indicated with asterisks. (A–D) At 24 hours p.f., the expected fraction of uninjected embryos begins to show brain necrosis, as indicated by decreased transparency in the head region (A). At this stage, embryos expressing products encoded by the wild-type (B) and maternal-effect mutant (C) alleles show no overt signs of necrosis. Expression of an engineered kinase dead mutant product (AurBK-R; [43]) results in an increased severity of brain necrosis in most embryos (D), showing that this product functions in a dominant negative manner. (A′–D′) Images at 48 hours p.f. of the same groups of embryos as in (A–D). Control uninjected *aurB^hi1045^* homozygotes now show a greater degree of necrosis in the anterior region, as reflected by increased darkening and a reduction of its size (A′). At this stage, *aurB^hi1045^* homozygotes injected with mRNA coding for wild-type (B′) and maternal-effect mutant (C′) Cei/AurB products begin to show brain necrosis, although generally to a lesser extent than uninjected mutant embryos. The degree of phenotypic rescue in embryos expressing wild-type product appears higher than that observed in embryos expressing the maternal-effect mutant product (less affected embryos in B′ are indicated by a white asterisk), which is consistent with the postulated hypomorphic nature of the maternal-effect mutant allele. By this stage, all embryos expressing AurBK-R protein exhibit some degree of necrosis (D′). Results shown are representative of duplicate experiments in embryos from two different clutches. Overexpression of neither maternal-effect mutant nor aurBK-R products (as well as wild-type product) did not have any observable effects on cell division at the cleavage stages (data not shown), possibly because of a temporal delay in protein product accumulation after mRNA injection at the one-cell stage.
(10.18 MB TIF)

Figure S5 Reduction of *aurB* function by a small molecule inhibitor phenocopies the *cei* mutant phenotype. Wild-type embryos treated beginning at 25 min p.f. with either carrier solvent (A) or ZM2 (B), fixed at 55 min p.f., and labeled to detect microtubules. The furrow corresponding to the second cleavage cycle (2^nd^, which initiates at 45 min p.f.) is either absent (B′) or truncated (B″). Furrow formation during the first cleavage cycle (1^st^, which initiates at 30 min p.f.) appears unaffected, likely because of a time lag for the effect of the drug. Embryos were synchronized by in vitro fertilization and are shown as animal views.
(4.45 MB TIF)

Figure S6 Expression of *cei/aurB* mRNA during embryogenesis. (A,C–F) Side views of fixed wild-type embryos processed through in situ hybridization using an antisense (A,C–F) and sense (B) *cei/aurB* probe. Developmental time points are: 8-cell (75 min p.f.), dome (4.3 hours p.f.), 75% epiboly (8 hours p.f.), tail bud (10 hours p.f.), 24-hour (24 hours p.f.).
(4.00 MB TIF)

Figure S7 Localization of Cei/AurB protein in *futile cycle* and maternally mutant *cellular island* embryos. Animal views of fixed embryos labeled to detect microtubules and Cei/AurB protein. (A–C) In *cei* mutant embryos, the mutant Cei/AurB protein becomes localized to the ends of FMA tubules that form in the shortened furrow. (D–F) In *futile cycle* mutant embryos, the FMA forms in an apparently normal manner and Cei/AurB protein is localized throughout the length of the furrow. (A′–C′) and (D′–F′) are higher power images of (A–C) and (D–F), respectively. The DAPI channel is included in (F,F′) to show the unfused pair of pronuclei characteristic of the *futile cycle* phenotype.
(10.06 MB TIF)
