# Supplementary figures and images for: The Maternal-Effect Gene cellular island Encodes Aurora B Kinase and Is Essential for Furrow Formation in the Early Zebrafish Embryo
Source: PLoS Genet. 2009 Jun 19;5(6):e1000518. doi: 10.1371/journal.pgen.1000518 (PMC2686166; doi:10.1371/journal.pgen.1000518)

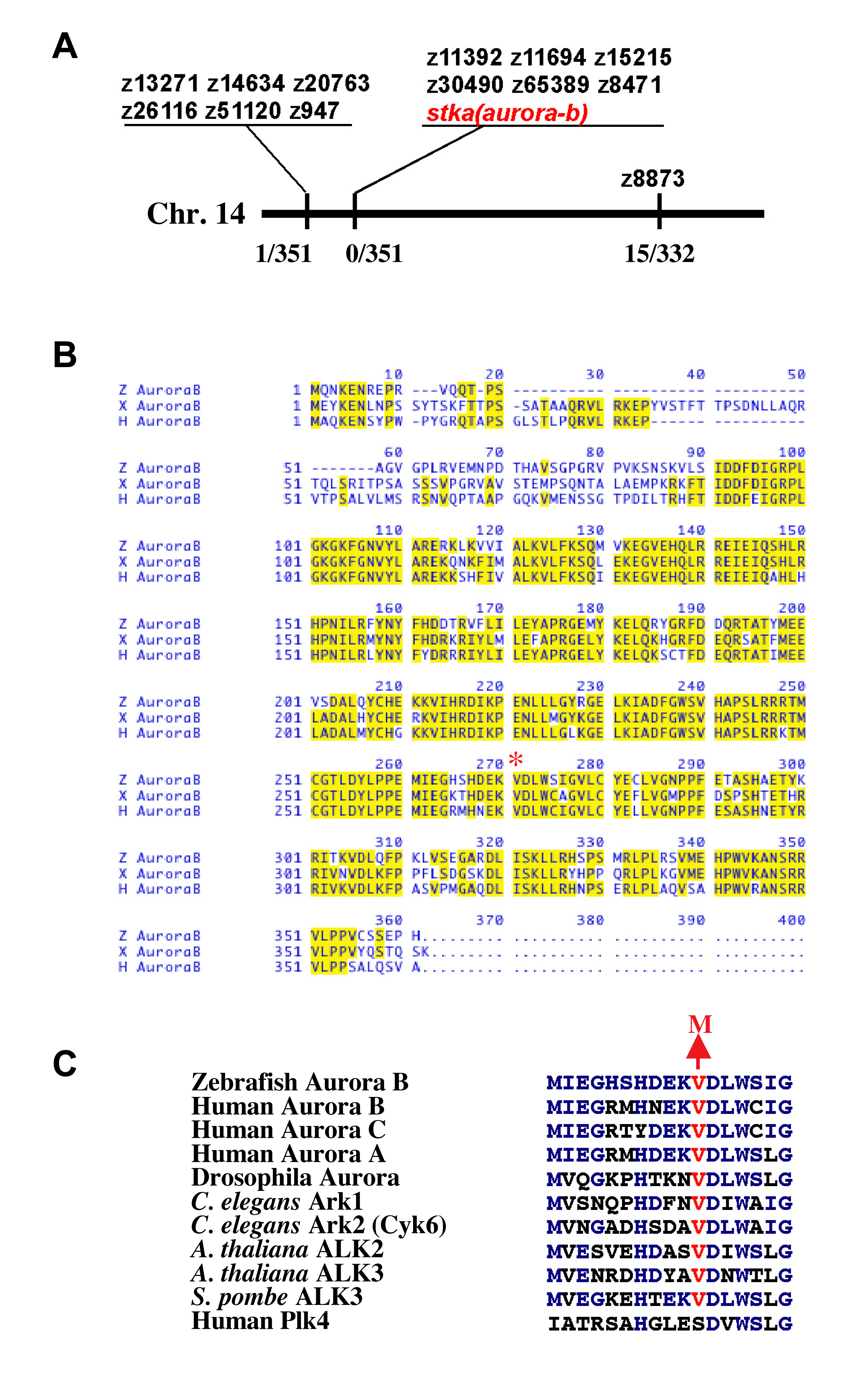

Supplement: Figure S1 — The cei mutation involves the substitution of a conserved amino acid in aurora B kinase. (A) Genetic linkage of the cei mutation to a region of Chromosome 14. Linked DNA markers and number of genetic recombinants over total number of analyzed meiotic events are indicated. (B) Sequence comparison of the zebrafish, Xenopus, and human Aurora B kinase protein. The red asterisk indicates the position of the amino acid substitution associated with the cei mutation. (C) Sequence conservation in the region of the identified amino substitution. The mutation results in a Valine→Methionine substitution at a position that is highly conserved amongst Aurora kinases but not conserved in the less related kinase Plk4. (5.77 MB TIF) [file pgen.1000518.s001.tif]

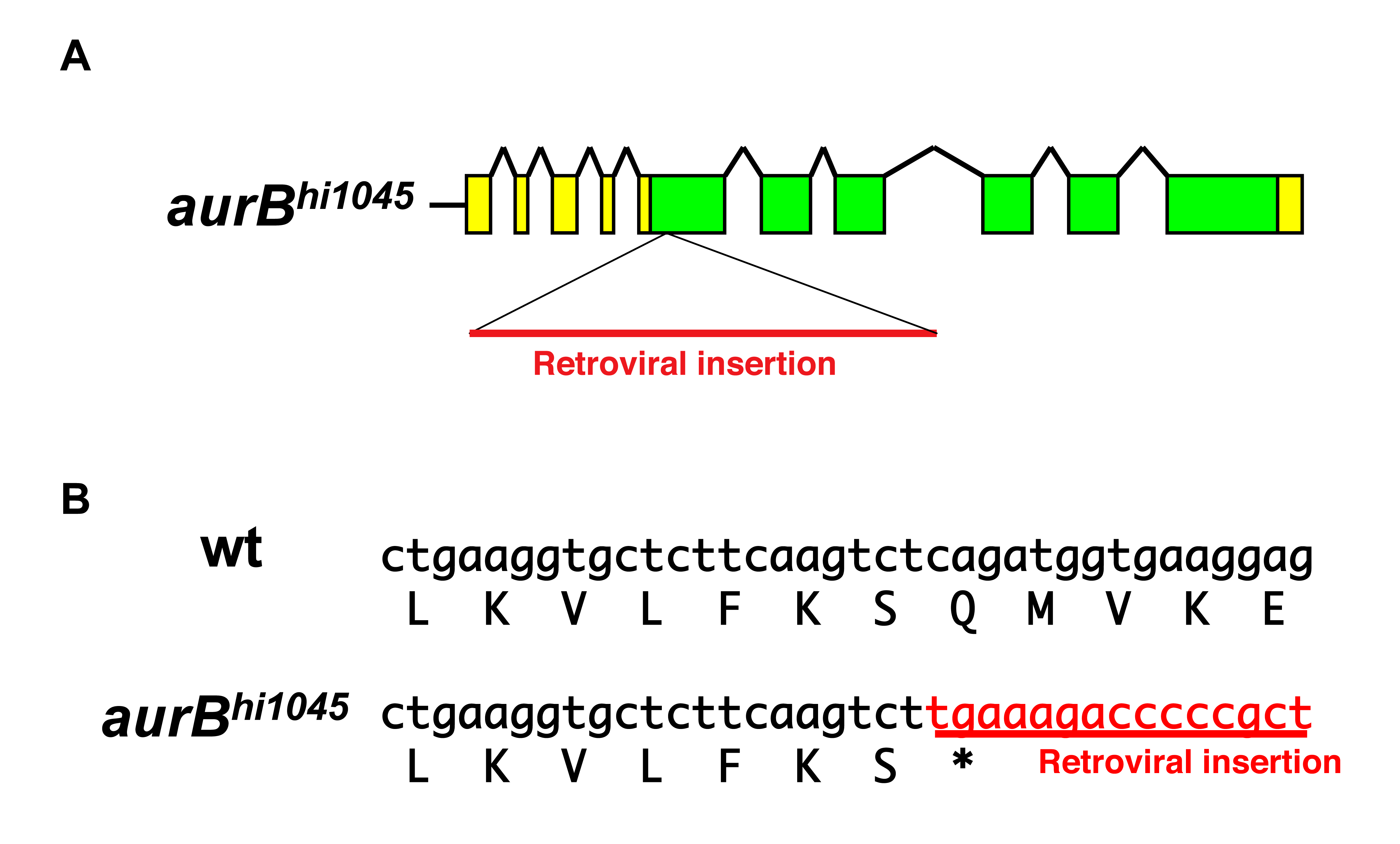

Supplement: Figure S2 — Retroviral insertion in the aurBhi1045 allele results in the truncation of zebrafish AurB. (A) Structure of the zebrafish aurB gene, depicting intron-exon boundaries, the kinase domain of the protein (in green) and the location of the retroviral insertion. (B) Sequence in the boundary region between the N-terminal portion of the AurB protein in the wild-type allele and in the aurBhi1045 allele, showing the premature stop codon caused by the retroviral insertion that results in the truncation of most of the kinase domain of AurB. (0.84 MB TIF) [file pgen.1000518.s002.tif]

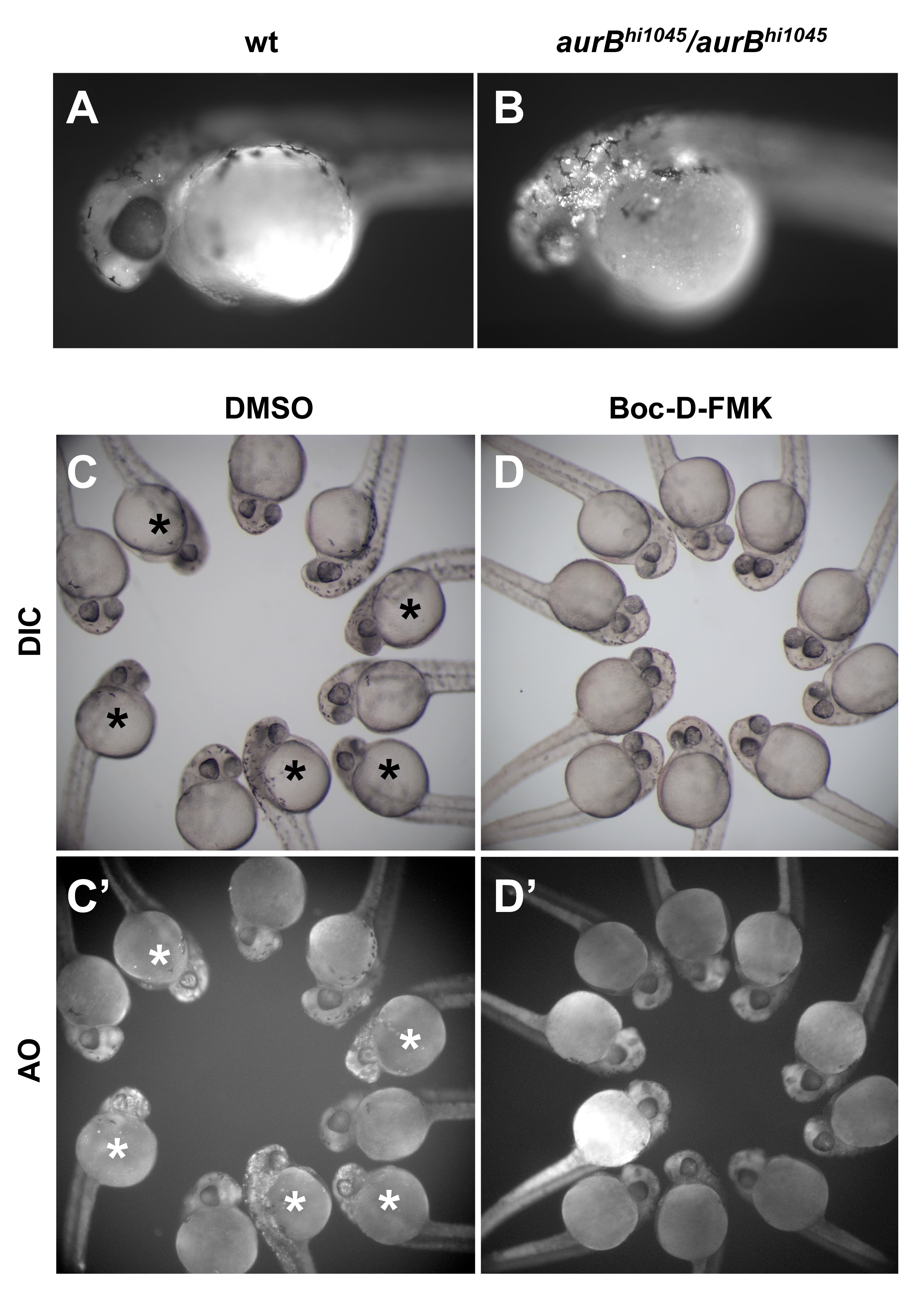

Supplement: Figure S3 — aurBhi1045 homozygotes exhibit an increase in cell apoptosis. (A,B) Exposure to the live cell apoptosis dye acridine orange brightly labels aurBhi1045 homozygotes (B), while control siblings only exhibit background levels of labeling (A). (C–D) Embryos from incrosses between aurBhi1045 heterozytes, 25% of which are expected to be aurBhi1045 homozygotes, were treated with either the caspase inhibitor Boc-D-FMK (D,D′) or control solvent (DMSO; C,C′). Embryos exhibiting the brain necrosis phenotype characteristic of aurBhi1045 homozygotes are indicated with an asterisk. Boc-D-FMK treatment prevents both the brain necrosis visible under standard microscopy (DIC; D, compare to C) and the accumulation of acridine orange labeled cells (AO; D′ compare to C′). (7.77 MB TIF) [file pgen.1000518.s003.tif]

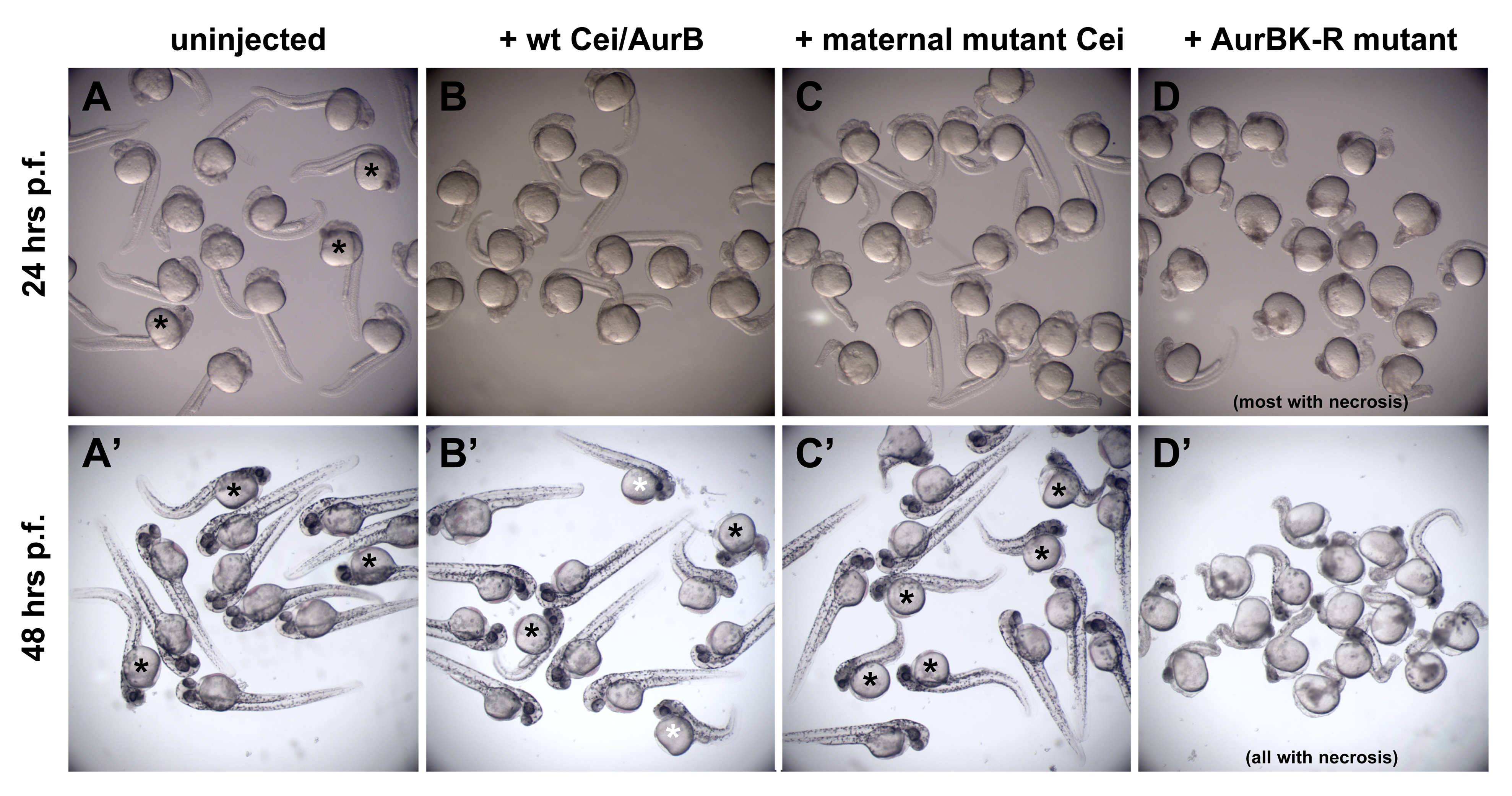

Supplement: Figure S4 — Expression of Cei/AurB products in aurBhi1045 homozygotes. mRNA was injected at the one-cell stage into embryos derived from incrosses of aurBhi1045 heterozygotes, so that an expected 25% of the progeny are aurBhi1045 homozygotes. In (A–C,A′–C′), embryos showing overt signs of necrosis are presumed to be aurBhi1045 homozygotes and are indicated with asterisks. (A–D) At 24 hours p.f., the expected fraction of uninjected embryos begins to show brain necrosis, as indicated by decreased transparency in the head region (A). At this stage, embryos expressing products encoded by the wild-type (B) and maternal-effect mutant (C) alleles show no overt signs of necrosis. Expression of an engineered kinase dead mutant product (AurBK-R; [43]) results in an increased severity of brain necrosis in most embryos (D), showing that this product functions in a dominant negative manner. (A′–D′) Images at 48 hours p.f. of the same groups of embryos as in (A–D). Control uninjected aurBhi1045 homozygotes now show a greater degree of necrosis in the anterior region, as reflected by increased darkening and a reduction of its size (A′). At this stage, aurBhi1045 homozygotes injected with mRNA coding for wild-type (B′) and maternal-effect mutant (C′) Cei/AurB products begin to show brain necrosis, although generally to a lesser extent than uninjected mutant embryos. The degree of phenotypic rescue in embryos expressing wild-type product appears higher than that observed in embryos expressing the maternal-effect mutant product (less affected embryos in B′ are indicated by a white asterisk), which is consistent with the postulated hypomorphic nature of the maternal-effect mutant allele. By this stage, all embryos expressing AurBK-R protein exhibit some degree of necrosis (D′). Results shown are representative of duplicate experiments in embryos from two different clutches. Overexpression of neither maternal-effect mutant nor aurBK-R products (as well as wild-type product) did not have any observ [file pgen.1000518.s004.tif]

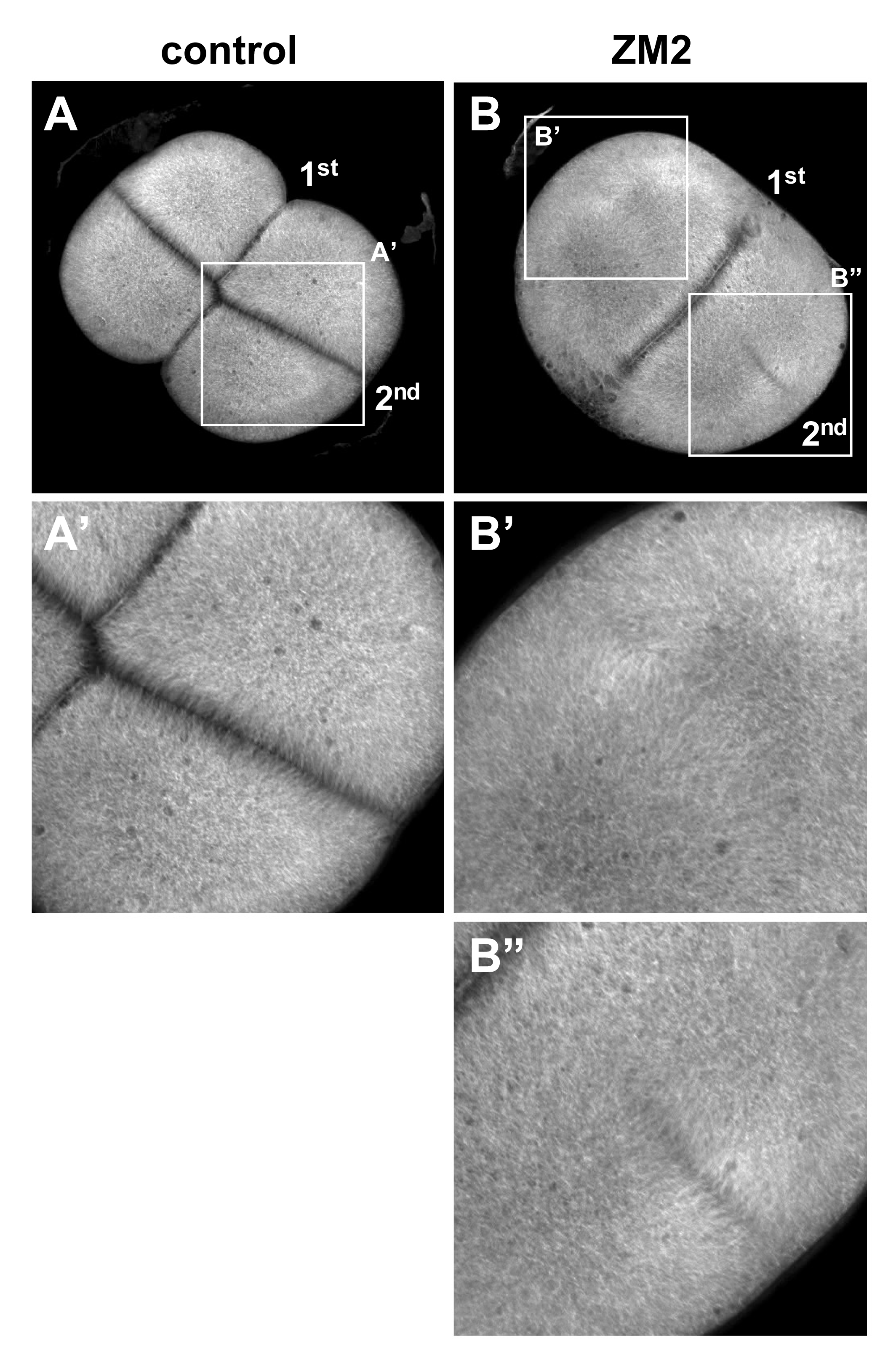

Supplement: Figure S5 — Reduction of aurB function by a small molecule inhibitor phenocopies the cei mutant phenotype. Wild-type embryos treated beginning at 25 min p.f. with either carrier solvent (A) or ZM2 (B), fixed at 55 min p.f., and labeled to detect microtubules. The furrow corresponding to the second cleavage cycle (2nd, which initiates at 45 min p.f.) is either absent (B′) or truncated (B″). Furrow formation during the first cleavage cycle (1st, which initiates at 30 min p.f.) appears unaffected, likely because of a time lag for the effect of the drug. Embryos were synchronized by in vitro fertilization and are shown as animal views. (4.45 MB TIF) [file pgen.1000518.s005.tif]

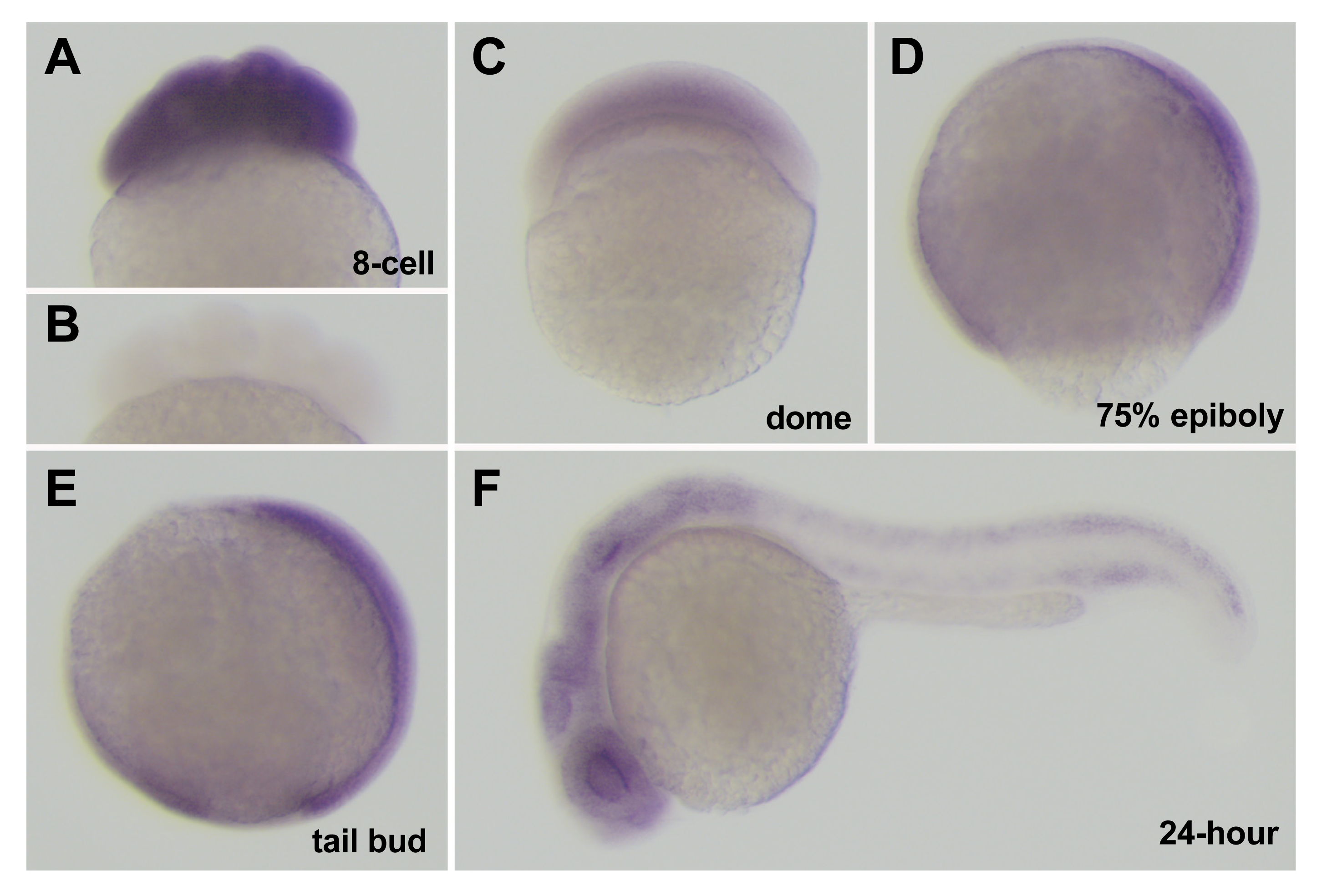

Supplement: Figure S6 — Expression of cei/aurB mRNA during embryogenesis. (A,C–F) Side views of fixed wild-type embryos processed through in situ hybridization using an antisense (A,C–F) and sense (B) cei/aurB probe. Developmental time points are: 8-cell (75 min p.f.), dome (4.3 hours p.f.), 75% epiboly (8 hours p.f.), tail bud (10 hours p.f.), 24-hour (24 hours p.f.). (4.00 MB TIF) [file pgen.1000518.s006.tif]

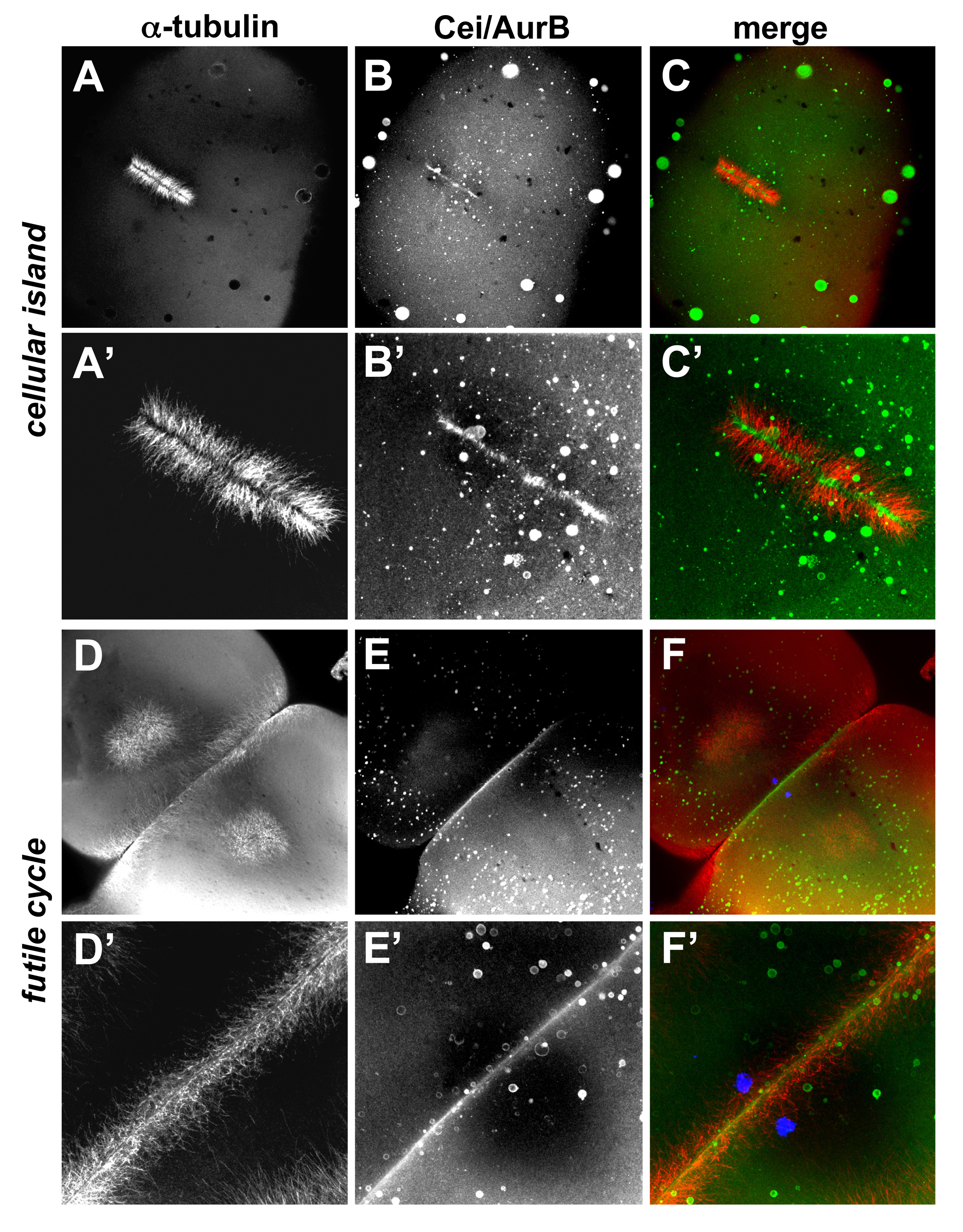

Supplement: Figure S7 — Localization of Cei/AurB protein in futile cycle and maternally mutant cellular island embryos. Animal views of fixed embryos labeled to detect microtubules and Cei/AurB protein. (A–C) In cei mutant embryos, the mutant Cei/AurB protein becomes localized to the ends of FMA tubules that form in the shortened furrow. (D–F) In futile cycle mutant embryos, the FMA forms in an apparently normal manner and Cei/AurB protein is localized throughout the length of the furrow. (A′–C′) and (D′–F′) are higher power images of (A–C) and (D–F), respectively. The DAPI channel is included in (F,F′) to show the unfused pair of pronuclei characteristic of the futile cycle phenotype. (10.06 MB TIF) [file pgen.1000518.s007.tif]
